# Dengue viruses – an overview

**DOI:** 10.3402/iee.v3i0.19839

**Published:** 2013-08-30

**Authors:** Anne Tuiskunen Bäck, Åke Lundkvist

**Affiliations:** 1 Department of Microbiology, Tumor and Cell Biology, Karolinska Institutet, Stockholm, Sweden; 2 Swedish Institute for Communicable Disease Control, Solna, Sweden; 3 Swedish International Development Cooperation Agency, Unit for Research Cooperation, Stockholm, Sweden; 4 IMBIM, BMC, Uppsala University, Uppsala, Sweden

**Keywords:** dengue virus, dengue fever, dengue hemorrhagic fever, dengue shock syndrome, flavivirus, vector-borne virus, arbovirus

## Abstract

Dengue viruses (DENVs) cause the most common arthropod-borne viral disease in man with 50–100 million infections per year. Because of the lack of a vaccine and antiviral drugs, the sole measure of control is limiting the Aedes mosquito vectors. DENV infection can be asymptomatic or a self-limited, acute febrile disease ranging in severity. The classical form of dengue fever (DF) is characterized by high fever, headache, stomach ache, rash, myalgia, and arthralgia. Severe dengue, dengue hemorrhagic fever (DHF), and dengue shock syndrome (DSS) are accompanied by thrombocytopenia, vascular leakage, and hypotension. DSS, which can be fatal, is characterized by systemic shock. Despite intensive research, the underlying mechanisms causing severe dengue is still not well understood partly due to the lack of appropriate animal models of infection and disease. However, even though it is clear that both viral and host factors play important roles in the course of infection, a fundamental knowledge gap still remains to be filled regarding host cell tropism, crucial host immune response mechanisms, and viral markers for virulence.

Dengue is an acute febrile disease caused by the mosquito-borne dengue viruses (DENVs), consisting of four serotypes (DENV 1 to 4), that are members of the *flaviviridae* family, genus flavivirus (1). All four DENV serotypes have emerged from sylvatic strains in the forests of South-East Asia (2).

DENV is presently the most common cause of arboviral disease globally, and all four serotypes of DENV can be found worldwide. More than 100 countries are endemic, primarily affecting 2.5 billion inhabitants in the tropical and subtropical regions (Fig. 1) as well as 120 million travelers to these regions every year (3). The World Health Organization (WHO) estimates an annual incidence of approximately 100 million infections, with approximately 500,000 people with dengue hemorrhagic fever (DHF) requiring hospitalization, a large proportion being children. DHF may develop into dengue shock syndrome (DSS) whereof the mortality rate is approximately 1–2.5%. Successful treatment of patients with DHF and DSS is labor intensive and expensive, but without proper treatment, fatality rates may exceed 20% (4).

**Fig. 1:**
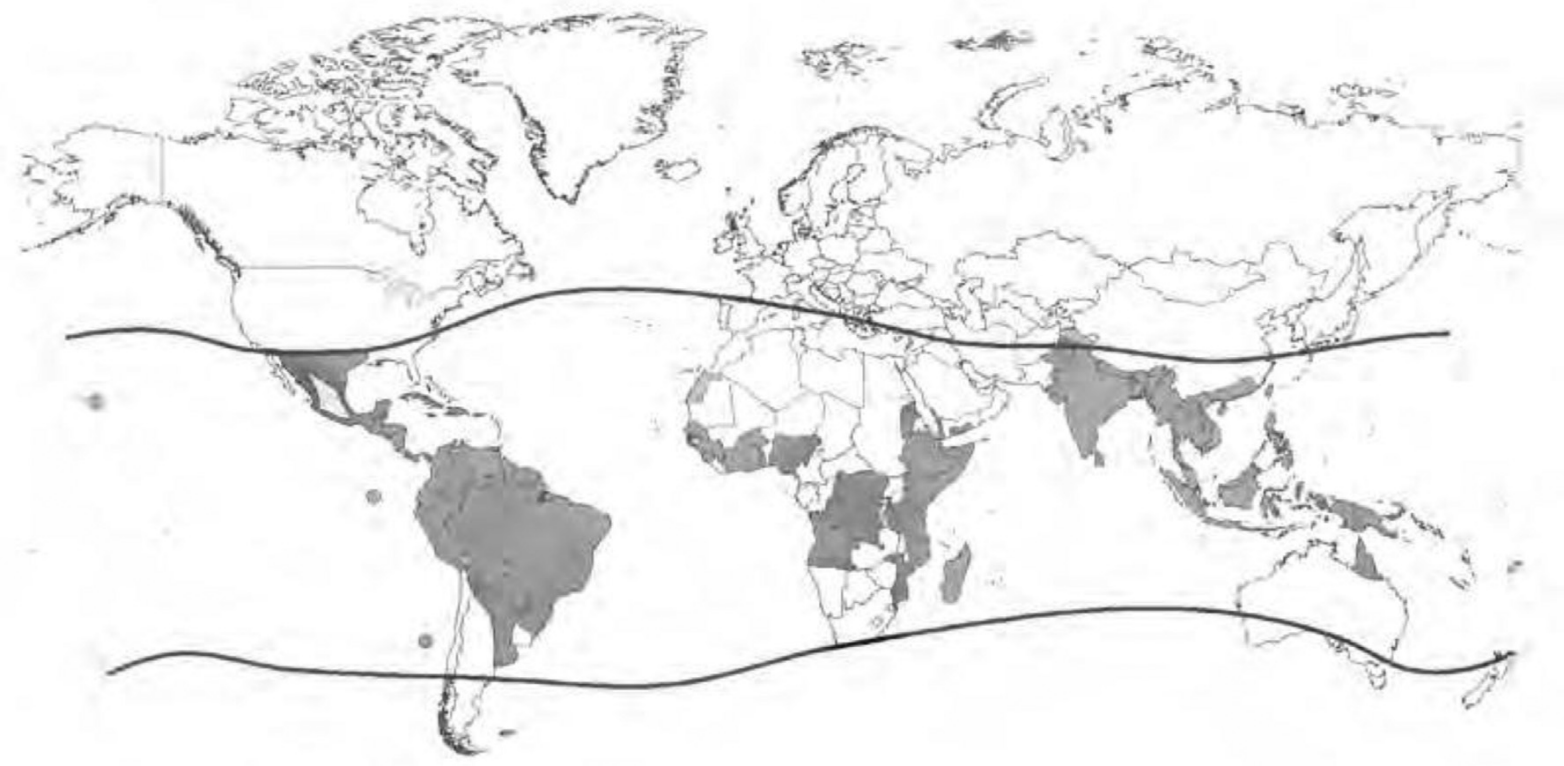
Dark-gray shading indicates countries/areas at risk of DENV transmission, 2008. The contour lines indicate the potential geographical limits of the northern and southern hemispheres for year-round survival of *Ae. aegypti*, the principal mosquito vector of DENVs. Reprinted by permission from WHO, Dengue: guidelines for diagnosis, treatment, prevention and control – New edition (2009), ^©^ 2009 (6).

The four DENV serotypes can cause a wide range of diseases in humans even though DENV infections may also be asymptomatic. The diseases range in severity from undifferentiated acute febrile illness, classical dengue fever (DF), to the life-threatening conditions DHF/DSS (5). Dengue illness was previously categorized on a I–IV grade scale, but a simplified categorization for dengue case classification has been proposed by WHO's Special Program for Research and Training in Tropical Diseases (TDR) in 2009 where DHF and DSS cases are grouped together as ‘severe dengue’ (group C) to avoid false-negative DHF/DSS diagnosis (6).

After an incubation period of 3–15 days (usually 5 to 8), classical DF begins with an abrupt onset of high fever. During the febrile phase, dehydration may cause neurological disturbances and febrile seizures in young children (6). The condition is self-limiting through debilitating illness with headache, retro-orbital pain, myalgia, arthralgia, petechiae rash, and leucopenia. A macular-papular recovery rash appears 3–5 days after the onset of fever, and it usually starts on the trunk before spreading peripherally (7). DF is sometimes referred to as ‘break bone fever’ due to its incapacitating symptoms with severe muscle and joint pain (8); or ‘seven-day fever’ since the symptoms usually persist for 7 days.

Early symptoms of DF and DHF are indistinguishable, but DHF is associated with hemorrhagic manifestations, plasma leakage resulting from an increased vascular permeability, and thrombocytopenia (<100,000 platelets/mm^3^). Thrombocytopenia is not necessarily restricted to severe dengue, and minor bleeding may occur in mild infections, which can be severe in those with peptic ulcer disease (9). Plasma leakage is characterized by haemoconcentration (haematocrit increase of 20%), development of ascites, or pleural effusion.

DSS is distinguished from DHF by the presence of cardiovascular compromise, which occurs when plasma leakage into the interstitial spaces results in shock. DSS is a fatal condition with mortality rates as high as 20% but can also be less than 1% in places with sufficient resources and clinical experience. Common clinical warning signs for DSS include a rapidly rising haematocrit, intense abdominal pain, persistent vomiting, and narrowed or absent blood pressure (5).

The number of reported dengue cases has increased dramatically since the 1980s due to several complex reasons (5, 10). The primary driving forces include rapid, unplanned urbanization combined with substandard living conditions, lack of vector control and surveillance, poor public health programs, international travel, and virus and vector evolution (3, 4). The contribution of climatic change is controversial, and it is not known to what extent this enhances the spread of mosquitoes, and indirectly the DENVs (3, 10, 11).

## Vector interactions

Transmission of DENVs is dependent on the vector mosquito *Aedes aegypti*, and to a lesser extent *Ae. albopictus*. The spread of DENVs mirrors the vectors’ geographical distribution underlining why mosquito density is an important parameter for predicting DENV epidemics (4)]. The female mosquitoes lay their eggs in artificial water containers such as tires, cans, and jars. Due to water requirements for breeding, mosquito densities peak during wet season, with the direct consequence of rising numbers of dengue cases. The *Ae. aegypti* mosquito is well adapted to an urban environment and is a highly competitive vector due to its anthropophilic nature. It thrives in close proximity to humans and is an intermittent feeder implying a high frequency of multiple host contacts during a single gonotrophic cycle. Thus, the female mosquito can infect multiple persons in order to complete a single blood meal. Protective clothing and mosquito-repellent sprays are essential to avoid DENV transmission since the *Aedes* mosquitoes are active during the day, minimizing the use of bed nets.

In general, *Ae. aegypti* is less susceptible to infection by DENV than *Ae. albopictus*, which could act as a selection mechanism for more virulent strains of DENV; the lower susceptibility would require a higher viral load in the human host in order to infect the mosquito. High viral titers in humans have been seen to be correlated to severe DHF/DSS. On the contrary, the secondary vector *Ae. albopictus* could transmit DENV strains that do not replicate to such high titers resulting in less clinically overt or severe disease. This scenario proposes that *Ae. albopictus* could function as a maintenance vector involved in the silent transmission of DENV during inter-epidemic periods. However, the susceptibility of the mosquito vector and transmission dynamics are also dependent on DENV strains, but the mechanisms underlying the inter-specific and inter-strain differences in vector susceptibility to DENV infection remains to be determined.

Once ingested by the mosquito, the DENV establishes a productive infection in the mosquito midgut, wherefrom the virus disseminates and replicates in other tissues. In order to be transmitted to a human (or non-human primate [NHP]) host during the next blood meal, the DENV must ultimately infect the salivary glands and be shed in the saliva. Vector competence is genetically determined, and genetic traits influencing both midgut infection and escape barriers have been mapped to various loci on the *Ae. aegypti* chromosomes (12).

Mosquito cell lines have been generated for studies of mosquito-borne viruses and can be used for field isolation of such viruses. *Ae. albopictus* cells have been used for isolation and identification of DENVs from a patient's blood (13, 14). Arboviral infection of mosquito cell culture yields high concentrations of virus and is characterized by a persistent infection as most mosquito cells are not killed by the infection (15).

In the continuing search for an effective vaccine and anti-dengue drugs; two measures to prevent DENV transmission are to reduce the vector population and to educate people in affected areas about basic protection measures. Anti-vector control programs include rigorous surveillance, spraying pesticides, genetically modified mosquitoes, minimizing potential breeding sites, and promoting decent housing and infrastructure. Personal protection includes protective clothing and anti-insecticide sprays.

Rapid, unplanned growth of urban centers in South-East Asian and South American countries combined with inadequate water supply and sewerage systems have dramatic consequences on the transmission of DENV (16, 17).

## The DENVs

### Replication cycle

DENV is an enveloped, single-stranded positive-sense RNA virus. The RNA genome consists of approximately 10,700 nucleotides and encodes a 3,411 amino acids long precursor polyprotein containing three structural proteins (capsid [C], precursor membrane [prM], and envelope [E]) and seven non-structural (NS) proteins (NS1, NS2A, NS2B, NS3, NS4A, NS4B, and NS5). The structural proteins are components of the mature virus particle whereas the NS proteins are expressed only in the infected cell and are not packaged to detectable levels into mature particles. The structural proteins are not involved in replication of the viral genome (18–20).

The open reading frame is flanked by two untranslated regions (5’ and 3’ UTR) of approximately 95–135 and 114–650 nucleotides, respectively. The 5’-end contains a type I cap, similar to cellular mRNA, and the viral RNA (vRNA) is translated by a cap-dependent initiation scanning the 5’-UTR. The 3’-end lacks a poly(A) tail but ends in a conserved stem-loop (SL) structure. Both the 5’- and 3’-UTRs are required for efficient translation and replication (21, 22). The UTRs have characteristic secondary structures that confer distinct functions and show high sequence conservation among different DENV serotypes. The 5’-UTR contains a large stem-loop (SLA) that is proposed to act as the promoter for the viral RNA-dependent RNA polymerase (RdRp) NS5 (23). Both the 5’- and the 3’-UTRs contain complementary Upstream AUG Regions (UAR) and cyclization sequences (CS) that hybridize in order to mediate genome cyclization and RNA synthesis (21).

The various steps in the flavivirus life cycle include 1) virions binding to cell-surface attachment molecules and receptors, and are internalized through endocytosis. 2) Due to the low pH of the endosome, viral glycoproteins mediate fusion of viral and cellular membranes, allowing disassembly of the virion and release of vRNA into the cytoplasm. 3) vRNA is translated into a polyprotein that is processed by viral and cellular proteases, and 4) the viral NS proteins replicate the genome RNA. 5) Virus assembly occurs at the endoplasmic reticulum (ER) membrane, where C protein and vRNA are enveloped by the ER membrane and glycoproteins to form immature virus particles. 6) Immature virus particles are transported through the secretory pathway, and in the acidic environment of the trans-Golgi network (TGN), furin-mediated cleavage of prM drives maturation of the virus. 7) Mature virus is released from the cell.

### Virus entry

Viral entry into the host cell is mediated by receptor-mediated endocytosis through an as yet unidentified cell-surface receptor. Candidate cellular receptors required for viral entry are various glycoproteins (i.e. heparin sulfates), dendritic cell-specific intercellular adhesion molecule-3-grabbing non-integrin (DC-SIGN), or a mannose receptor (24–26). The human C-type lectin-like molecule CLEC5A has been suggested to act as a critical macrophage receptor for DENV and has been described as a proinflammatory receptor for DENV that contributes to lethal disease in mice (27, 28). There is a general consensus that the viral E glycoprotein affects host cell receptor binding, viral entry, and is a major target for humoral immunity. The E protein is composed of three domains: domain I, domain II harboring the fusion peptide at its distal tip, and domain III responsible for receptor-binding activity. In the mature state, E exists as a homodimer with the fusion peptide inaccessible. Low-pH–induced trimerization exposes the hydrophobic fusion peptide in a manner consistent with membrane fusion mediated by class II fusion protein (29). Mutations of residues constituting the ligand pocket at the interface of domain I and II alter the required pH threshold and affect virulence (30). There are two potential asparagine (N)-linked glycosylation sites at positions Asn-67 and Asn-153, whereof the former is unique for DENVs and the latter is conserved in most flaviviruses (31). The glycosylation pattern differs according to DENV serotype and even among different strains, as well as the cells in which the virus is propagated. The degree and position of N-linked glycans affect the antigenic properties of DENV (32–34).

Upon internalization, the acidic pH in the endosome triggers a conformational change in the E protein mediating membrane fusion. The viral nucleocapsid is released into the cytoplasm whereupon the virus uncoats and releases the genome (31). The input positive-strand vRNA is translated into a single polyprotein, which is cleaved into the individual structural and NS proteins. The input strand translation is followed by a switch from translation to synthesis of a negative-strand intermediate, which serves as a template for new positive-strand vRNA. Multiple rounds of translation produce high levels of viral proteins that together with vRNA are assembled into progeny virions (35).

DENV infection induces intracellular membrane alterations in the cytosol forming vesicle packets (VPs) or smooth membrane structures (SMS) where the viral replication complex (RC) accumulates (36, 37). The induction of membrane structures may serve as a scaffold for anchoring the viral RC. The C-terminal regions of C, prM, and E contain hydrophobic amino acids that serve as signal sequences for insertion of the remaining protein into the ER membrane (38). An ER signal peptidase together with the viral NS2B-NS3 protease cleaves the structural proteins and NS1 protein into individual membrane-bound proteins (39–41). The NS3 protein acts, together with its cofactor NS2B, as the viral serine protease needed for polyprotein-processing through its N-terminal end (42, 43). This heterodimer protein complex cleaves on the cytoplasmic side of the ER membrane at the junctions between NS2A-NS2B, NS2B-NS3, NS3-NS4A, and NS4B-NS5, as well as on the internal sites within C, NS2A, NS3, and NS4A.

The C-terminal end of the NS3 protein has three enzymatic properties: a 5’ RNA-triphosphatase (RTP), a nucleoside triphosphatase (NTPase), and a helicase. NS3 forms a complex with NS5 and assists in viral replication through unwinding of RNA and dephosphorylation prior to 5’-end capping. The remaining NS proteins are cleaved by the viral serine protease NS3 that requires NS2B as a cofactor for catalytic activity (43). However, a host cell signal peptidase mediates post-translational modifications on the NS4A-4B proteins (44).

The NS1 is a glycoprotein with two glycosylation sites that are conserved among flaviviruses. It is synthesized in the ER as a hydrophilic monomer but exists as a more hydrophobic homodimer. The NS1 dimer is transported to the Golgi apparatus where it undergoes carbohydrate trimming (45). The role of NS1 in virus replication is unknown but is believed to facilitate viral infection and DENV pathogenesis (46). NS1 is in addition secreted from infected cells (sNS1) and has been shown to be immunologically important (47, 48). Antibodies raised against sNS1 proteins have been proposed to cause endothelial dysfunction due to cross-reactivity to host proteins and endothelial cells (49). Data indicate that sNS1 could be an important modulator of the complement pathway and is proposed to protect DENV from complement-dependent neutralization in solution (47).

The small hydrophobic proteins NS2A, NS4A, and NS4B are less well characterized. Recent findings propose an inhibitory role in interferon (IFN)-mediated signal transduction. Their hydrophobic nature potentially implicates them in proper localization of viral proteins and vRNA during replication and virion assembly. Formation of DENV-induced cytoplasmic membrane structures are believed to be an arrangement of the NS4A protein.

The largest NS protein encoded in the DENV genome is the NS5 protein, approximately 103 kDa big. The NS5 protein has three major functional domains: the N-terminal S-adenosyl methionine methyltransferase (MTase), the nuclear localization sequences (NLS), and the RdRp activity in its C-terminal domain. The MTase spans amino acid residues 1 to 239 and is responsible for guanine N-7 and ribose 2’-*O*-methylations required for the capping of the DENV genome. The cap structure is recognized by the host cell translational machinery. The NLS (residues 320–405) interacts with the NS3 viral helicase and is recognized by cellular factors, allowing protein transport to the nucleus. The NS5 polymerase domain RdRp (residue 273–900) is responsible for synthesizing new vRNA genomes (43).

Prior to secretion of new viral particles, the third structural protein (pr)M is processed into the mature M protein in the TGN by furin host protease (50). It is believed that prM protects the E proteins from pH-induced reorganization and premature fusion during secretion; hence, the maturation event is necessary for infectivity (51–53).

### Laboratory diagnosis

Since laboratory-based dengue diagnosis is often unavailable at the time of care, the preliminary diagnosis relies on a combination of travel history and clinical symptoms. Travel history provides key information that can rule out other potentially life-threatening diseases since the incubation period of DENV is less than 2 weeks (54). A confirmed diagnosis for a DENV infection is established by culture of the virus, polymerase-chain reaction (PCR), or serologic assays. There are, however, limitations with each test, and detection is based on different virological markers, namely infectious virus, vRNA, and DENV-specific antibodies, respectively.

Culturing the virus requires an acute patient serum with sufficient levels of virus, and the period when DENV can be successfully isolated in patient serum is short. Viremia peaks before the onset of symptoms, hence virus levels might drop significantly once the patient seeks medical care. Furthermore, rising levels of antibody interfere with virus culture already within a day or two after the subsidence of fever. Apart from sample collection limitations, practical considerations limit the use of this method. Culture of the virus is both time- and labor intensive; infectious patient material must be kept cold, and a bio-safety level 3 laboratory is required, necessitating professional training of the personnel. These requirements limit the use of this diagnostic tool, especially in rural areas (4).

The *Ae. albopictus* cell line C6/36 (CRL 1660, ATCC) is commonly used to isolate DENVs from patient material. Specimens that may be suitable for virus isolation include acute phase serum; plasma or washed buffy coat from the patient; autopsy tissues from fatal cases, especially liver, spleen, lymph nodes, and thymus; and mosquitoes collected in nature (55). Detection of vRNA from serum, plasma, or cells with PCR is based on DENV-specific oligonucleotide primers, and is fast and robust, although sensitive only in the very early stages of disease (54). PCR is particularly useful in situations when virus culture has not been successful but nevertheless depends on sample collection during the symptomatic phase.

The third laboratory diagnostic option is not based on direct detection but on the presence of anti-DENV antibodies. Thus, it is not hindered by the limitations of virus culture and PCR, and the timing of sample collection can be more flexible. The acute anti-DENV IgM antibody response lasts for a couple of weeks after infection and the IgG antibodies for several years. The immunoglobulins (Ig) are not easily inactivated and do not have the same strict requirements for low temperature as infectious virus specimen. The assay techniques are relatively simple and there are commercial diagnostic kits available, whereof the assays based on IgM detection are the most commonly used in routine diagnostics (4). The major drawback with serological tests is the considerable risk for false-positive results due to potential cross-reactivity with other flaviviruses, for example, vaccination against Yellow fever virus (YFV) (56).

Due to the drawbacks of serological methods to reliably diagnose acute infections, alternative methods based on the detection of the viral NS1 protein have been developed. NS1 can be found both membrane-associated inside the host cell and in a soluble, secreted form. The amount of secreted NS1 in patient serum correlates with viremia and DENV pathogenesis (46, 57–60), and the NS1 protein is detectable in serum by enzyme-linked immunosorbent assay (ELISA) from the first day of fever up to 9 days post-infection (46, 61–63). NS1-based ELISAs have become an important diagnostic tool for acute samples in which IgM is not detectable and where PCR is not available. Several commercial NS1 antigen kits are available and are widely used in endemic as well as non-endemic countries. The sensitivity varies from 63% to 94% (58, 62, 64, 65), and it depends on sample time-point, DENV serotype, and if it is a primary or secondary DENV infection (66).

### Vaccines

Unlike flaviviruses such as YFV, Japanese encephalitis virus (JEV), and Tick-borne encephalitis virus (TBEV), no licensed vaccine exists for dengue. Vaccination must protect against all four serotypes without predisposing for antibody-dependent enhancement (ADE) and has proven difficult to design. Nearly 80 years of vaccine-related research and development have passed, and over 25 unique DENV vaccine candidates have been tested in clinical trials during the past decade.

To be safe, a dengue vaccine must be functionally tetravalent, eliciting simultaneous protection against all four DENV serotypes. Hence, vaccination cannot proceed in an analogous sequential manner, and herein lies the greatest obstacle (67–69). Live attenuated vaccines can induce durable humoral and cellular immune responses that mimic natural infection (70). However, the viral replication must be discrete to preclude the development of significant illness. A reasonable range of viremia for a live attenuated vaccine is believed to be approximately 10^1^-10^2^ infectious units/mL (71) compared to high levels of viremia upon natural infection that can be 10^5^-10^7^ infectious units/mL (59).

It is expected that a live attenuated vaccine would be successful and require only a single dose since the vaccine against YFV is based on a live attenuated virus. However, it is more likely that booster immunizations will be required based on results from clinical trials using tetravalent formulations of live vaccine candidates aimed at eliciting neutralizing antibodies (72–75) The obvious challenge is when and how to boost; infectivity and immunogenicity in NHP models have not always clearly predicted the outcome of human trials (76, 77). Vaccination compliance may also be lower with a multi-dose vaccination strategy, especially in regions where resources are scarce, and at the same time where the need for a vaccine often is the most acute.

Currently, there are several dengue vaccine candidates at different stages of preclinical or clinical development. The most advanced clinical development stage is a candidate developed by Sanofi Pasteur (CYD-TDV), which is under evaluation in phase II and phase III clinical studies. Phase III efficacy studies of CYD-TDV are currently underway in 31,000 children and adolescents in 10 countries in Asia and Latin America. These multi-center studies in a variety of epidemiological settings will be important to obtain data regarding efficacy and safety, and will shed further light on the relationship between vaccine-induced immune responses and protection against clinical dengue disease (73).

There are in addition other live-attenuated, subunit, and DNA vaccine candidates at earlier stages of clinical development. Other technological approaches include viral-vectored and virus-like particle vaccines, which currently are being tested in preclinical studies. It is hoped that clinical trials evaluating novel recombinant subunit proteins, DNA, and vectored vaccines would be initiated in the coming years. These approaches could be part of a prime-boost strategy, or stand-alone (78). The use of different types of vaccines depends on the purpose of vaccination and target group reflecting the disease setting. In endemic areas, there is an urgent need for routine immunization against dengue for infants and young children aged 1–3 years. A dengue vaccine would be coordinated with current childhood immunization schedules. Due to the socioeconomic status of many endemic countries, this type of vaccine ought to be inexpensive. In contrast, a protective vaccine for international travel, seasonal work personnel, and military staff that visit or work in DENV endemic areas are more tolerant to increased cost. Vaccination in this case will need to be rapid.

Hence, the different requirements for a dengue vaccine vary according to target group and their specific needs (life-long immunity or temporal protection), and efficient antiviral drugs would be a useful complement for protection and/or treatment. In addition, antiviral drugs would be more potent in an outbreak situation than a vaccine when there is no time to complete a multi-dose immunization schedule spanning 6 months or more.

### Treatment and therapeutic approaches

Currently, vector control, regarded as both expensive and ineffective, is the only method for disease prevention (79, 80). In the absence of available vaccines and antiviral drugs against DENV infection, specific treatment for dengue patients consist primarily of supportive care including bed rest, antipyretics, and analgesics. Urgent resuscitation with intravenous fluids to replace lost intravascular volume in DSS patients is a prerequisite; Ringer's lactate has been shown to be effective in moderately severe dengue, and starch or dextran have been suggested for more severe cases (81). Aspirin and other salicylates should be avoided due to plasma leakage (6).

The design of novel therapeutic approaches for dengue disease has focused on the various stages of the viral replication cycle. The conformational changes of the E protein and its interaction with prM or M have been a major interest. These transition states present opportunities for antiviral targeting of the entry, assembly, or maturation steps of the virus life cycle. Antiviral peptides have been designed and tested for blocking of both DENV and West Nile virus (WNV) entry with positive results, indicating that antiviral peptides could be a promising form of DENV therapy. Targeting of mature virus entry into host cells is an extremely promising candidate since delivery of target compounds into the host cell during stages of fusion and maturation is significantly more challenging.

Another approach to inhibit the structural changes of the E-prM protein interactions has been to synthesize peptides mimicking the pr peptide of the M protein, thereby preventing membrane fusion and release of newly synthesized virions. The viral protease is another interesting target for antiviral discovery, since proteases are common to most viruses and generally important for efficient replication. Protease inhibitors for hepatitis C virus (HCV) may eventually be further developed to inhibit the DENV protease NS2B-NS3.

Nucleoside analogues are usually prodrugs that need to be converted to their antiviral nucleotide metabolite forms. Ribavirin (1-β-d-ribofuranosyl-1*H*-1, 2, 4-triazole-3-carboxamide) possesses broad spectrum antiviral activity and is used in combination with IFN to combat HCV infection. Ribavirin depletes the nucleotide pool and thereby indirectly affects capping and polymerase activities of both cellular and viral proteins. In addition, ribavirin causes a more error-prone replication of several viral genomes. Despite successful *in vivo* results with several RNA viruses, ribavirin has a cytostatic effect in DENV-infected cells and has not been effective in animal models.

Nucleic acid–based therapies offer various alternatives. RNA interference (RNAi) is thought to protect the host from viral infections by degrading the extraneous genetic material such as vRNA. It has been used in therapeutic approaches for several infectious diseases, tumors, and metabolic disorders. Small interfering RNA (siRNA) treatment reduces viral load of WNV in mice, but there are several obstacles yet to overcome; the RNA of flaviviruses are resistant to RNAi since replication occurs in reorganized ER membrane packets. In addition, HCV replication was found to be stimulated by the RNAi machinery.

Another nucleic acid–based antiviral approach is antisense DNA or RNA decoys, for example, phosphorodiamidate morpholino oligomers (PMOs). These compounds act by forming a stable, sequence-specific duplex with RNA, thereby blocking access to target RNA by biomolecules required for replication. PMOs targeting the translation initiation site of DENV RNA, the 3’ UTR, 5'SL, and 3'CS were effective in reducing the viral load in various cell lines. These compounds meet most of the requirements for an anti-DENV therapeutic; non-toxic, cheap, easy to administer, stable for months at variable temperatures, but remain to be tested in animal models.

Sulfated polysaccharides have been investigated for anti-DENV activity, although inconsistency in the activity results indicates that they need to be further tested both *in vitro* and *in vivo* (24).

The processing of *N*-linked oligosaccharides in the ER is important for viral glycoprotein maturation, and inhibition of glucosidase-mediated trimming affects the replication cycle of several enveloped viruses. DENV production was inhibited in mouse neuronal cells by two ER α-glucosidase inhibitors, castanospermine (CST) and deoxynojirimycin. CST was effective against all four serotypes in human hepatoma cells and prevented mortality in DENV-2–infected mice. This effect was restricted to DENV, not being observed against other flaviviruses such as WNV and YFV. A third ER α-glucosidase inhibitor, *N-*nonyl-deoxynojirimycin, inhibits DENV-2 infection in BHK-cells. These results with α-glucosidase inhibitors are encouraging and should be investigated further *in vivo*.

Nitric oxide (NO) is generated by macrophages, monocytes, dendritic cells (DCs), and neutrophils; the same cells that are supposed to be the main sites of replication for DENV. *In vitro* assays have revealed that NO specifically affects the viral RdRp activity, suggesting possible viral targets of NO during DENV infection (82).

Hence, there are multiple options for designing novel therapeutics for dengue disease. However, the main concern with most therapeutic approaches is that they are not validated for inhibitory effects on all four DENV serotypes. In addition, several studies have not been examined in an animal model, and several reported antivirals have been tested at only one time point, pre- or post-infection in tissue culture systems, and therefore need to be subjected to more diverse regimes, and different cell types.

## Risk factors for severe dengue

**Fig. 2:**
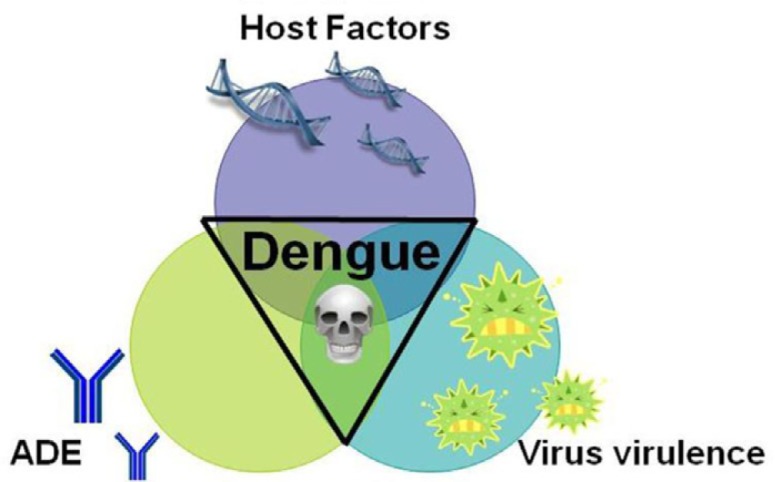
The complex interplay of risk factors for severe dengue disease can be illustrated as a triangular interplay dominated by the three main risk factors: host factors, preexisting DENV-specific antibodies mediating antibody-dependent enhancement (ADE), and intrinsic virus features influencing strain virulence. The exact contribution of each risk factor may vary from case to case.

DENV pathogenesis remains a challenging jigsaw puzzle with many pieces missing to understand the complex interplay of viral and host factors. Despite intensive research, it is not well understood. The severity of DENV infection is modulated by multiple risk factors such as age (83, 84), the genetic background of the host (85, 86), viral serotype (83, 87) and genotype (88, 89), and secondary DENV infection by a heterologous serotype (85, 90–93). Finally, the virus serotype and genotype also influence the symptomatic picture of disease and outcome (Fig. 2). These observations were initially based on epidemiological findings, but accumulating laboratory and experimental data have contributed to the recognition of DENV virulence as an important risk factor.

### Host genetics

Apart from the influence of viral genetic determinants, the host's genetic background with varying polymorphisms might have important consequences for disease susceptibility (94). Improvements in high-throughput genotyping of genetic polymorphisms have permitted a genome-wide approach to the investigation of host genetic susceptibility. However, most studies have not attempted functional trials to try to link genetic association with any process in disease pathogenesis.

Indirect evidence of the host's genetic importance has been derived from Cuban dengue epidemics where a reduced risk for DHF/DSS was observed in those with an African ancestry compared to those with European ancestry (3). The Cuban observations coincide with the low susceptibility to DHF reported in African and Black Caribbean populations (86, 94). It is interesting that despite the circulation of DENV in 19 African countries, there are only sporadic reports of DHF cases (94).

In order to better understand these population differences, the polymorphic HLA genes have been among the most studied candidates for genetic associations with DHF/DSS. Several serological studies of HLA class I alleles have been performed in ethnically and geographically distinct populations, and positive correlations of various HLA class I alleles with susceptibility to DHF have been found. A significantly higher frequency of HLA class I alleles A*31 and B*15 have been found in Cuban individuals with symptomatic DENV infection compared to asymptomatic controls, who showed an elevated frequency of HLA II alleles DRB1*07 and DRB1*04. The DRB1*04 was also the most frequent allele associated with resistance to DHF in the Mexican Mestizo populations of the Americas. Since the Mexican Mestizo population and the Cuban population share the same Amerindian genetic background, it is possible that the identification of the same HLA class II allotype could explain the association to dengue disease protection.

A case-control study in ethnic Thai cases also reported the association of HLA class I alleles (A2, A*0207, B46, B51) with different clinical outcomes. A similar study in a Vietnamese population confirmed the association with polymorphism of the HLA class I loci and DHF suslceptibility. The same study also found that polymorphisms in the HLA-DRB1 allele are not associated with DHF susceptibility, highlighting the findings in the Amerindian populations.

The number of studies on polymorphisms within genes other than the HLA loci remains low. Variants of the vitamin D receptor and the FcγRIIA gene are associated with resistance to severe dengue. In addition, an allelic variant of the DC-SIGN1 coding gene CD209 is believed to protect against DHF (94).

### Host health and age

An increased association between severe dengue and bronchial asthma, diabetes mellitus, peptic ulcers, and sickle cell anaemia has been observed (94, 95). However, the impact of dengue on chronic diseases and other pathogens needs to be further investigated.

Primary infections are supposed to cause mild disease in children, compared to secondary infections that tend to lead to severe dengue. In South-East Asia, DHF/DSS is predominantly an illness in children. The greater relative prevalence of DSS in children relative to adults is believed to be due to the intrinsically more permeable vascular endothelium in children (94). There is no clear consensus; studies conducted in South American countries have reported similar (84) as well as contradictory results indicating that adults are the most affected (94).

### Autoimmune responses in dengue virus infection

Anti-DENV antibodies can cross-react to host proteins and endothelial cells, and this could enhance the endothelial dysfunction observed in DHF/DSS. Antibodies against the viral surface E protein cross-react with plasminogen and have been associated with bleeding in acute DENV infection, and anti-DENV NS1 antibodies cross-react with host proteins and endothelial cells (49, 94). In addition, immune activation markers (e.g. IL-6, IL-8, TNFα, IFNγ, and complement components 3A and 5A) together with altered platelet, DC, monocyte, and T-cell functions suggest that immune responses to various DENV components could contribute to autoimmune processes resulting in DHF/DSS (94).

### Antibody-dependent enhancement

A secondary infection by a heterologous DENV serotype is an important risk factor for developing DHF/DSS. The explanation lies within the cross-reactive antibodies raised after a primary DENV infection (90, 92, 94). Serotype-specific antibodies confer life-long immunity to the homologous serotype, whereas cross-protection against heterologous serotypes last for 3–4 months. Beyond this time period of cross-protection, the preexisting antibodies of sub-neutralizing concentration will instead cross-react with the heterologous virus facilitating viral infection of FcγR-bearing cells. This phenomenon is known as ADE (94). The limited cross-protection between the four DENV serotypes has allowed them to coexist in the same or overlapping geographical areas. Thus, their antigenic uniqueness has implied an evolutionary advantage (96).

Low-affinity/Low-affinity, sub-neutralizing antibodies and DENV form virus-antibody immune complexes that bind to Fcγ-receptors on monocytes. The net result will be a larger number of infected cells compared to the primary infection when there were no cross-reactive antibodies present, or compared to earlier after the primary infection when antibody levels are high enough to achieve neutralization of the heterologous virus. Hence, the viral biomass will be larger during a secondary DENV infection compared to during a primary DENV infection.

*In vitro* studies indicate that non-neutralizing antibodies against the viral prM protein can potentially mediate ADE. These anti-prM antibodies are in addition non-neutralizing even at high concentrations (97, 98). The proposed hypothesis for prM-mediated ADE is based on the fact that the viral prM protein needs to be cleaved to render the virus infectious. Hence, immature virus particles that would otherwise be non- or less-infectious are rendered infectious in combination with anti-prM antibodies that mediate ADE to infect new host cells (97).

The time interval between heterotypic DENV infections is another parameter influencing the magnitude of ADE; a longer interval between heterologous DENV infections causes higher DHF/DSS ratios (85). The differences in DENV genotype could influence the pathogenic consequences, but a contributing risk factor is the progressive loss of heterotypic neutralizing antibodies (99). The time effect of DENV-specific antibodies can be seen in DENV-immune mothers and their infants. Before the age of 3–4 months, the maternally derived DENV-specific antibodies confer protection against a DENV infection. However, primary infections in infants aged between 4 and 12 months of age run a higher risk of developing severe dengue due to maternally derived non-neutralizing antibodies. The risk of severe dengue decreases after the age of 1 year as the concentration of cross-reactive antibodies declines (94, 100, 101).

A higher viral burden elicits a greater host inflammatory response and increased plasma levels of proinflammatory cytokines. Secondary DENV infections and severe disease in DHF/DSS patients have elevated serum levels of IL-2, IL-6, IL-8, IL-10, IL-13, IL-18, IFNγ, TNFα, and MCP-1 (102–108). Thus, an increased infected cell mass would stimulate T-cell and cytokine responses that are proportional to the antigenic stimulus. This hypothesis is consistent with the observations that a high initial viremia or high NS1 concentrations in blood during secondary infections are associated with DHF/DSS (46, 59, 109, 110).

Accumulating evidence questions whether ADE of infection alone is sufficient to explain DHF/DSS (59). Severe dengue with plasma leakage can occur in primary infection without ADE. In addition, by the time plasma leakage occurs, viral titers are several logs below peak levels, and there are patients with high viral titers that do not develop plasma leakage (59, 109, 111). Thus, increased viremia alone is not the direct cause of plasma leakage and other mechanisms are involved in the cytokine storm. Furthermore, ADE is not a useful correlate of disease risk (112, 113).

### Dengue virus virulence

ADE has dominated as the explanatory model for severe dengue disease in secondary infections. However, evidence for ADE in humans is indirect and controversial results against ADE exist (112, 114). Many parts of the world have become hyperendemic, implying that all four serotypes of DENV co-circulate in the same country, with the consequence that secondary infections are common scenarios. Epidemiological data also indicate that not all secondary infections cause DHF/DSS, and that there are even cases of tertiary and quaternary DENV infections (115). Studies from Thailand report that 0.08%–0.8% of dengue hospitalizations may be caused by tertiary and quaternary DENV infections (67). In Cuba, 17.5% of the total DHF dengue cases were caused by third or fourth infections (116).

The four DENV serotypes 1–4 diverge at ∼30% across the polyprotein (117), but each serotype also consists of phylogenetically distinct ‘subtypes’ or ‘genotypes’, which have different geographical distributions (94). The hypothesis that some DENV genotypes have greater virulence and epidemic potential than others was introduced during the 1970s around the same time that the ADE phenomenon was coined (118–120). However, in contrast to the ADE hypothesis, experimental evidence for increased virulence was for long absent and, therefore, primarily based on epidemiological observations. Recent work has shed light on this question and confirmed what Rosen, et al. proposed almost four decades ago (118, 119).

There have been specific geographic examples of the appearance of DENV genotypes correlating to DHF/DSS epidemics. The appearance of a South-East Asian DENV-2 strain in the Americas in 1981 resulted in the sudden emergence of DHF/DSS cases. It turned out that DENV-2 could be subdivided into a variety of genotypes, minimally Asian and American. The Asian genotype is more virulent and more likely to result in DHF/DSS than the American genotype even after a secondary infection (121, 122). Viruses to the South-East Asian DENV-2 lineage replicate to higher titers in human DCs than American genotype viruses. It was also seen that the South-East Asian genotype infects and disseminates to the head tissue of *Ae. aegypti* mosquitoes more rapidly and in a greater proportion compared to the American genotype viruses (121).

The emergence of group B subtype III DENV-3 strain in Sri Lanka in 1989 is another example of clade replacement correlating with an increase in DHF/DSS (88, 123, 124). As for the South-East Asian DENV-2 strain, the invasive DENV-3 strain replicated to higher levels in mosquitoes and disseminated to the head tissue more readily than the displaced, native DENV-3 strain from Sri Lanka (125). Both traits likely enhanced the capacity to spread and displace endemic strains.

Based on the examples given, one hypothetical mechanism for increased virulence suggests that highly pathogenic DENV strains have been selected for enhanced ability to replicate in key human targets, such as macrophages and DCs (94, 126). Thus, virulent DENVs would produce more viruses per cell, resulting in higher viremia and inflammatory response, than with a low pathogenic strain (94, 127).

The second hypothesis for increased virulence proposes that strains associated with severe DHF/DSS better escape neutralization by the presence of serotype cross-reactive antibodies in the semi-immune host compared to strains associated with DF (128). Enhancement of virus replication following heterologous infection may favor coexistence of multiple serotypes. If such enhancement also results in increased transmission, DENVs from different serotypes would benefit from prior and concurrent circulation of several serotypes in the same location (96).

It is still not known if the tendency of certain genotypes to cause severe disease results from greater intrinsic virulence, or if greater virulence is a result of enhanced infectivity in the presence of heterologous antibodies, or a combination of the two. Determining whether DENVs differ in virulence, as well as identifying the genetic basis of such differences, is of fundamental importance.

## Immune responses and dengue virus pathogenesis

DENV infection is a systemic and dynamic disease with a wide clinical spectrum. Gross pathological findings in cases of DHF or DSS include hemorrhages in the skin, subcutaneous tissues, gastrointestinal tract, and heart (129). Hemorrhage, dilatation and congestion of vessels, and edema of arterial walls are commonly found, and hemorrhagic manifestations in other organs combined with fluid accumulations in body cavities may be substantial (130, 131).

However, the underlying mechanisms of vascular leakage and hemorrhage are not well characterized. Elevated plasma levels of pro-inflammatory and vasoactive cytokines before and at the time of plasma leakage in patients with DHF suggest that excessive cytokine production (a ‘cytokine storm’) induce vascular permeability. Available data propose that the outcome of a DENV infection depends on a balance between favorable and unfavorable immune responses; the former providing control of viral replication, whereas the latter enhancing inflammatory and vascular permeability. The lack of reliable immunological markers for either protective or pathological immune responses to DENV and the lack of a suitable animal model for dengue disease hamper the understanding of dengue pathogenesis. Insights into the immune response against DENV infection rely primarily on clinical and epidemiological studies.

### Tropism

Identification of the primary target cells of DENV replication has proven to be extremely difficult. Existing data are based on virus detection by immunohistochemical (IHC) analysis with antibodies against viral structural proteins, or by *in situ* hybridization to the positive-strand vRNA. However, it is difficult to prove direct infection of specific target cells by these methods as a positive signal could be due to virus endocytosed or phagocytosed by uninfected cells. Detection of negative-strand vRNA and/or DENV NS proteins would provide much stronger evidence of active DENV replication.

After inoculation by an infected mosquito, the initial round of viral replication is believed to occur in the subdermal Langerhans DCs (132–135). These infected cells become activated and migrate to draining lymph nodes (136). The activated DCs elicit a robust IFNα/β and TNFα response together with a strong pro-inflammatory response to limit contiguous spread (133). Viral replication continues in still undefined cells in the lymph node. There is a general consensus that candidate cell types belong to the macrophage-monocyte lineage. Autopsies and human biopsies confirm that cells from the mononuclear phagocyte linage probably are the primary targets of DENV infection following initial dissemination from the local skin site. Infiltrating mononuclear cells in affected tissues have been shown to contain DENV antigen (137, 138), and DENVs can occasionally be isolated from peripheral blood leukocyte fractions (139). Similar observations have been made in rhesus macaques where DENV was recovered from leukocyte-rich tissues such as regional lymph nodes, systemic lymphatic tissues, and disseminated skin sites.

Infection is amplified within the lymph nodes and viremia can be detected when the infectious virus enters the circulation via the efferent lymphatic system and thoracic duct. Circulating monocytes in the blood are believed to be infected due to the viremia facilitating spread to secondary visceral organs where macrophages within the spleen, liver, and bone marrow are infected (140–145).

There has been limited and inconsistent dissemination to solid organs (146); DENV antigen has been detected in lymphocytes (140, 147), hepatocytes (147–150), endothelium (140, 147, 151, 152), and cerebral neurons and astrocytes (147, 152). There are in addition other studies with contradicting results where the same tissues have been examined without any detected DENV antigen (140, 150, 151).

A further controversy surrounds the role of endothelial cells as the target for DENV infection. Severe dengue disease is characterized by systemic endothelial dysfunction accompanied by vascular leakage, even though destructive vascular lesions are generally absent in fatal cases (83). Primary human endothelial cells and human endothelial cell lines are permissive for DENV infection (47, 153), but endothelial infection, however, does not seem to be required for severe pathologic changes in individual tissues (154). Their contribution *in vivo* remains to be established.

The presence of DENV antigens in various organs and cell types suggest that the host receptor(s) is broadly distributed. Host receptors for DENV are believed to include mannose binding protein, heparan sulfate, chondroitin sulfate, and DC-SIGN (26, 155, 156). Following DENV infection natural antibodies (IgM), complement, and possibly NK cells control the initial levels of viremia and to certain extent tissue dissemination. Upon recognition by cytotoxic T lymphocytes, infected cells are targeted by the cellular immune system (discussed below).

### The humoral immune response

The humoral immune response is hypothesized to be vital for controlling DENV infection and dissemination, and infection with one serotype provides long-lasting protection to that specific serotype (homotypic immunity). Subsequent infection by another serotype results in short-lived protection (heterotypic immunity), and may eventually be harmful and increase the risk of severe dengue disease. The transient nature of heterotypic immunity is believed to be due to cross-reactive viral E protein–specific antibodies which are protective above a certain concentration threshold (157).

The principal targets of the antibody response to DENV infection in humans are the prM, the E structural proteins, and the NS1 protein. Weak antibody responses to other NS proteins, for example, NS3 and NS5, have also been detected (158, 159). Neutralizing antibodies are directed against the viral E protein and inhibit viral attachment, internalization, and replication within cells. There are multiple epitopes residing within each of the three E domains (160, 161), but not all are equally accessible for antibody binding due to the dimeric conformation of the E protein on the virion surface, and its tight packing in the mature form (162–164).

Domain III of the E protein, which contains the putative host receptor-binding site, is the most variable in amino acid sequence between serotypes. As a result, antibodies specific for this domain show the greatest degree of serotype specificity (165). However, mutations in domain III of the E protein are common for escaping neutralizing antibody (166, 167). Loss of an effective neutralizing antibody response due to sequence variation has also been detected for the C and NS2B proteins (168, 169).

Antibodies against DENV may also bind to complement proteins and promote their activation. Anti-prM and/or E protein antibody–mediated complement fixation to virions can inhibit viral infection (170). As for other host immune responses to dengue, complement involvement may also be pathological. Complement activation is a feature of severe dengue and is temporally related to plasma leakage. This suggests that complement activation constitutes a major factor in the pathogenesis of dengue hemorrhagic shock (171, 172). Increased complement activation at endothelial cell surfaces could contribute to the vascular leakage, and the viral protein NS1 is proposed to be a modulator of the complement pathway. By promoting efficient degradation of C4 to C4b, NS1 may protect DENV from complement-dependent neutralization in solution (47).

### The cellular immune response

In addition to the humoral immune response, cellular immune responses are also crucial in dengue pathogenesis. The DENV can infect both CD4+ T-cells and CD8+ T-cells (173), and similar to DENV-specific antibodies, the cellular immune responses can be either protective or harmfully reactive. DENV-specific T-cells respond with a diverse set of effector functions, including proliferation, target cell lysis, and the production of a range of cytokines. CD4+ T-cells produce IFNγ, TNFα, TNFβ, interleukin (IL)-2, and CC-chemokine ligand 4 (CCL4; also known as MIP1β) which may contribute to pathogenesis (174). The production of T helper type-2 cytokines, such as IL-4, is less common (175–178). In uncomplicated DENV infections, relatively more CD8+ T-cells are present resulting in lower levels of IFNγ and TNFα (179). CD8+ T-cell clones specific for DENV partially protect mice from lethal DENV challenge (180). The role of T-regulatory cells is unclear in dengue, but there is a study suggesting they are functional and expand in acute DENV infection (181).

Following primary infection, both serotype-specific and serotype cross-reactive memory T-cells are generated. Upon secondary exposure, both the protective and cross-reactive memory T-lymphocytes are activated and the non-protective memory T-cells will augment infection (182). Activated memory T-cells recognize both conserved and altered peptide ligand epitopes. The antigen sequence differences depend on the specific DENV epitope but will nevertheless affect the quality of the effector T-lymphocyte response. This in turn modifies the immunological repertoire and is suggested to be involved in the development of plasma leakage (183). A full agonist peptide will induce a full range of T-cell responses including production of multiple cytokines (e.g. IFNγ, TNF, and CCL4) and lysis of the infected cell. A partial agonist peptide, that is, one which varies at one residue, will cause cross-reactivity in memory T-cells and induce a skewed functional response, involving production of some cytokines but little of other cytokines and inefficient cell lysis. Thus, because of sequence diversity between DENV serotypes, the memory T-cells (and B cells) that are re-activated during a secondary DENV infection may not have optimal avidity for the epitopes of the new infecting virus. The ‘memory’ of the primary DENV infection alters the immune response to the secondary infection influencing the clinical outcome. There is a correlation between the level of T-cell responses and disease severity (184, 185). The phenomenon of low affinity for the current infecting serotype but a high affinity for a past infection with a different serotype is referred to as *Original Antigenic Sin*, and is the net effect of an altered balance between a protective and pathological outcome (185, 186). The pattern of antibody/T-cell responses in secondary DENV infections is also influenced by the sequence and interval between DENV infections (67, 116, 187–190). As for the ADE scenario, memory T-cell responses exhibiting serotype cross-reactive proliferative activity decades after the primary infection could potentially alter the balance from a protective immune response toward an improper and non-protective immune response. Interestingly, most of the identified CD4+ and CD8+ T-cell epitopes reside in the NS3 protein, which represents only ∼20% of the DENV amino acid coding sequence (179).

### Cytokines in dengue pathogenesis

Viral recognition by the host cell occurs immediately upon virus entry to raise an appropriate antiviral response. Two main families of pathogen recognition receptors mediate DENV sensing; the extracellular/endosomal toll-like receptors (TLRs) (191, 192) and the cytoplasmic receptor family of DExD/H box RNA helicases (e.g. retinoic acid inducible gene 1 [RIG-1] and melanoma differentiation-associated gene-5 [MDA5]) (193). Binding to a TLR leads to activation of two families of transcriptional factors: the interferon regulatory factors (IRFs) and the nucleic factor-kappa B (NF-kB). These signaling cascades activate production of IFNα/β and proinflammatory cytokines that stimulate maturation of DCs and elicits an antiviral response (133, 194).

DENV is believed to primarily infect cells of the DC/macrophage/monocyte lineage via receptor-mediated endocytosis and/or enhanced uptake via antibody–virus complexes attached to Fc-gamma receptors (FcγR) (195). The exact mechanisms behind DHF/DSS are not understood, but the consensus is that infected cells and activated endothelial cells produce TNFα (196, 197), and NO (198, 199), increasing vascular wall permeability (200). The coincidence of severe disease manifestations with abatement of fever and virus control suggests that the symptoms may be a consequence of the immune response against the virus rather than virus-induced cytopathology. Consistent with this hypothesis is the increased levels of many different cytokines that have been observed in DENV infection (201). Elevated serum levels of cytokines and chemokines include IL-2 (202–204), IL-6 (205, 206), IL-8 (207), IL-10 (109, 202), IL-13, IL-18 (105), IFNγ (102, 103), TNFα (102, 205, 208, 209), and monocyte chemotactic protein-1 (MCP-1) (210). Furthermore, these cytokines are associated with secondary infections and severe dengue implying immunopathogenesis. However, it is not fully understood how these cytokines cause malfunction of vascular endothelial cells leading to plasma leakage. A Th1-type response is linked to recovery from acute infection, whereas a Th2-type response is associated with exacerbation of infection and a poor clinical outcome. Patients with non-severe dengue predominantly have a Th1-type response. Cross-regulation of Th1 and Th2 is primarily mediated by IL-10 and IFNγ, respectively (211). In addition, activated macrophages recruit CD4+ T-cells that produce human cytotoxic factor (hCF), which in turn induces a cytokine cascade that leads to a Th1-type or Th2-type response. Levels of hCF can be elevated in severe dengue cases and hCF autoantibodies protect against severe disease (201). As the severity of the illness increases, the response shifts to a Th2-type response, characterized by secretion of IL-4, IL-5, IL-6, IL-10, and IL-13. Infections primarily eliciting a humoral immune response induce a higher expression of Th2-related cytokines (201, 211).

Pathological features of hemorrhagic dengue are increased capillary permeability in the absence of morphological damage to the capillary endothelium, altered number and functions of leukocytes, increased haematocrit and thrombocytopenia. Thrombocytopenia is accompanied by plasma leakage and deregulated coagulation, and the latter is likely to be mediated by cytokines, for example, TNFα (212)]. Increased levels of IL-6 and IL-8 are associated with deregulated coagulation and fibrinolysis in dengue (213, 214). Thus, it is believed that indirect effects of virus infection render the vascular wall permeable. Secreted TNFα from activated, infected cells promotes increased endothelial permeability and increases the expression of adhesion molecules on endothelial cells (215–218), whereas increased IL-10 levels correlate to reduced levels of platelets and reduced platelet function (109, 219). This could contribute to the development of bleeding complications. Extensive plasma leakage into various serous cavities of the body, including the pleural, pericardial, and peritoneal cavities, may result in profound shock.

The immunopathogenesis of dengue has to a large extent been correlative in nature describing temporal associations between cytokine concentrations and the clinical events of plasma leakage. There is a need to identify causal immunopathogenic mechanisms compared to the abundance of descriptive studies of dengue pathogenesis. It is worth noting that other infectious diseases and inflammatory disorders with elevated cytokine levels are not accompanied by increased vascular permeability as seen in severe dengue. Thus, the challenge is to identify the key elements of the host immune response that are causally linked to papillary permeability from those that constitute the normal host immune response.

## Animal models of dengue virus infection and disease

The pathogenesis of DENV infections is, despite intensive research, not well understood. Many fundamental questions in dengue pathogenesis are difficult to address due to the lack of appropriate animal models of infection and disease. No non-human species naturally exhibits the more severe forms of dengue disease that mimic human DF, DHF, and DSS, and this has hampered the development of a suitable animal model.

### Non-human primates

NHPs are the only vertebrates apart from humans known to be naturally infected by DENV. However, the viral strains isolated from NHPs are genetically distinct from those infecting humans indicating that these transmission cycles diverged a long time ago (2). In addition, NHPs do not develop hemorrhagic fever, but only a mild infection. Animal models of DENV infection were however initially limited to NHP despite the fact that NHPs only develop mild viremia without severe clinical symptoms. Attempts to induce dengue-like disease in other animals failed and the suboptimal NHP models remained the only available model for a long time. Studies in rhesus macaques revealed no abnormalities in haematocrit or prothrombin time, and only a minority of animals displayed a limited decrease in platelet counts. Thus, DENV infection in macaques has been unable to offer any major insights into the molecular mechanisms of DENV pathogenesis. In contrast, NHPs are widely used in vaccine testing as they are capable of developing neutralizing antibodies in response to DENV infection (220).

### Mouse models

Rodents are susceptible to DENV infection although they do not exhibit disease similar to that in human DENV infections. To compensate for these limitations, murine models have relied on mouse-adapted DENVs that appear to be attenuated with respect to human infection. New mouse models have been developed to render the mice more susceptible to infection but none have been able to mimic DENV-specific immune responses.

Mouse models based on intracranial inoculation of DENV have for long been used in parallel to studies in NHPs. Mice usually exhibit a neurovirulent phenotype quite unlike human dengue disease, and succumb to intracranial infection. Multiple efforts have generated a diverse set of mouse models for DENV infection, each with distinct advantages and disadvantages (220). The various approaches can be divided into four groups: 1) Immunocompetent mouse models, 2) Severe combined immunodeficient (SCID)-tumor transplant mouse models, 3) Humanized mouse models, and 4) Interferon-deficient mouse models. Consequently, the various models have contributed in different ways to understanding mechanisms underlying DENV pathogenesis and immunity, and in the development of antiviral drugs and vaccines.

The unimpaired nature of the immune system of immunocompetent mice renders them poorly susceptible for DENV infection. Despite the lack of clinical symptoms, these models have been used extensively to study other aspects of DENV pathogenesis (212, 221–225). In addition, immunocompetent mouse models of DENV infection have been popular for drug and vaccine development studies since the intact immune system is valuable for assessing vaccine immunogenicity. Clinical and neurological disease can be induced by high-dose infection and/or intracranial injection, as well as mouse-adapted DENV strains that render mice more susceptible to DENV infection (226, 227). However, the relevance of observations made with manipulated DENV strains should be interpreted with caution regarding their relevance to wild-type infections.

Measurable viremia is a desired trait when studying DENV infection, and a common strategy to render the mouse more susceptible to DENV infection is to manipulate the mouse in one or several ways. Transplanted human tumor mass that provides a replication site for infectious DENVs have been applied in SCID mice. Since SCID mice lack an adaptive immune system, DENV infection has been successfully established within the transplanted cells and even some dengue disease features have been reproduced (228–230). However, it is unclear how any insights into pathogenesis might apply to human pathogenesis since viral replication is restricted primarily to the transplanted cells.

An improved strategy based on the SCID-tumor model is ‘humanized’ SCID mice that are irradiated to destroy the haematopoietic progenitors in the bone marrow, prior to transplantation with human CD34+ haematopoietic stem cells. The result is an adaptive immune system consisting exclusively of human cells, with certain parts of the innate system being humanized as well (231, 232). The advantage with this model is the increased susceptibility to clinical DENV isolates without adaptation to mice, and the mice display some signs of human disease. The exact cell types in which DENV replicates in humanized mice remains to be identified. In addition, the difficulties with the humanized mouse model are multiple, such as the genetic variation in stem cell donors, the hardship to generate sufficient numbers of humanized mice, variability in the degree of human cell engraftment, and radiation sensitivity of SCID-mice (233). A limitation of crucial importance is the lack of lymph nodes that support a human immune system (234–236), as well as the lack of a robust immune response in these humanized mice; both fluctuations in the elicited immune response as well as poor reproducibility (237, 238). Human-specific cytokines required for human cell development and survival are not successfully generated, and there is a low and variable level of T-cell-dependent antibody responses (239, 240). Thus, humanized mouse models are time-consuming and labor-intensive and, therefore, not optimal for most drug and vaccine development purposes. These new models may eventually have the potential to answer important and fundamental questions concerning infection of human cells *in vivo*, but there are still major issues to be resolved regarding stem cell development in order to obtain a functional and intact adaptive immune system.

Another way to increase the susceptibility of mice to DENV infection is to generate mice deficient in the IFN pathway (241, 242). The IFN system is a potent suppressor of DENV replication, and mice lacking the IFN-α/β and –γ receptors (AG129 mice) can be lethally challenged without intracranial inoculation (241). The AG129 mouse strain exhibits viral replication in relevant peripheral cell types such as macrophages and DCs in spleen and lymph node (243), hepatocytes, and myeloid cells in the bone marrow (154). A variety of DENV isolates have been used and the clinical symptoms are either neurological disease or a hemorrhagic shock-like vascular permeability syndrome depending on the virus isolate and route of inoculation (241, 243–248). Consequently, AG129 mice have been used for numerous studies, including tropism, pathogenesis, immune protection and enhancement, as well as for antiviral drug testing and vaccine development (241–243, 246, 247, 249, 250). However, the genetically deficient background does not make the AG129 mouse suitable for genetic studies since it would be time-consuming to introduce a third knock-out gene.

## Conclusions

The DENVs are old viruses that have re-emerged during the latter half of the 20th century. Regarded as a tropical fever disease affecting more than two thirds of the world's population, dengue is also the main cause after malaria of tropical fever among travelers and ranks as the most important mosquito-borne viral disease in the world. The lack of potent antiviral drugs and an effective vaccine results in ∼500,000 individuals, mainly children, being hospitalized with severe dengue every year and causes tremendous economic losses for both households and whole nations. The pathogenesis of the DENVs are not well understood, partly due to the absence of good animal models. Effective vector control measures are the sole weapon against dengue today, while we are hoping for improved diagnostics, clinical treatment, and an effective vaccine.

## References

[1] Westaway EG, Brinton MA, Gaidamovich S, Horzinek MC, Igarashi A, Kaariainen L (1985). Flaviviridae. Intervirology.

[2] Wang E, Ni H, Xu R, Barrett AD, Watowich SJ, Gubler DJ (2000). Evolutionary relationships of endemic/ epidemic and sylvatic dengue viruses. J Virol.

[3] Guzman MG, Kouri G (2002). Dengue: an update. Lancet Infect Dis.

[4] WHO (1997). Dengue haemorrhagic fever. Diagnosis, treatment, prevention and control.

[5] Gubler DJ (2002). Epidemic dengue/dengue hemorrhagic fever as a public health, social and economic problem in the 21st century. Trends Microbiol.

[6] WHO (2009). Dengue: guidelines for diagnosis, treatment, prevention and control – New ed.

[7] Henchal EA, Putnak JR (1990). The dengue viruses. Clin Microbiol Rev.

[8] Rush B, Garrison-Morton (1789). An account of the bilious remitting fever, as it appeared in Philadelphia in the summer and autumn of the year 1780. *Medical inquiries and observations*.

[9] Tsai CJ, Kuo CH, Chen PC, Changcheng CS (1991). Upper gastrointestinal bleeding in dengue fever. Am J Gastroenterol.

[10] Thomas SJ, Strickman D, Vaughn DW (2003). Dengue epidemiology: virus epidemiology, ecology, and emergence. Adv Virus Res.

[11] Tun-Lin W, Burkot TR, Kay BH (2000). Effects of temperature and larval diet on development rates and survival of the dengue vector Aedes aegypti in north Queensland, Australia. Med Vet Entomol.

[12] Ooi E-E, Gubler DJ, Hanley KA, Weaver SC (2010). Dengue virus-mosquito interactions. Frontiers in dengue virus research.

[13] Singh KR, Paul SD (1969). Isolation of dengue viruses in Aedes albopictus cell cultures. Bull World Health Organ.

[14] Pavri KM, Ghosh SN (1969). Complement-fixation tests for simultaneous isolation and identification of dengue viruses, using tissue cultures. Bull World Health Organ.

[15] Stalder J, Reigel F, Flaviano A, Koblet H (1981). Infection of the Aedes albopictus cell clone C6/36 with Semliki Forest virus. Experientia.

[16] McCall P, Kittayapong P (2006). Control of dengue vectors: tools and strategies. Report of the ScientificWorking Group on Dengue.

[17] McCall PJ, Lenhart A (2008). Dengue control. Lancet Infect Dis.

[18] Pang X, Zhang M, Dayton AI (2001). Development of dengue virus replicons expressing HIV-1 gp120 and other heterologous genes: a potential future tool for dual vaccination against dengue virus and HIV. BMC Microbiol.

[19] Pang X, Zhang M, Dayton AI (2001). Development of Dengue virus type 2 replicons capable of prolonged expression in host cells. BMC Microbiol.

[20] Alvarez DE, Lodeiro MF, Filomatori CV, Fucito S, Mondotte JA, Gamarnik AV (2006). Structural and functional analysis of dengue virus RNA. Novartis Found Symp.

[21] Gamarnik A, Hanley KA, Weaver SC (2010). Role of the dengue virus 5’ and 3’ untranslated regions in viral replication. Frontiers in dengue virus research.

[22] Padmanabhan R, Strongin AY, Hanley KA, Weaver SC (2010). Translation and processing of the dengue virus polyprotein. Frontiers in dengue virus research.

[23] Yu L, Nomaguchi M, Padmanabhan R, Markoff L (2008). Specific requirements for elements of the 5’ and 3’ terminal regions in flavivirus RNA synthesis and viral replication. Virology.

[24] Chen Y, Maguire T, Hileman RE, Fromm JR, Esko JD, Linhardt RJ (1997). Dengue virus infectivity depends on envelope protein binding to target cell heparan sulfate. Nat Med.

[25] Tassaneetrithep B, Burgess TH, Granelli-Piperno A, Trumpfheller C, Finke J, Sun W (2003). DC-SIGN (CD209) mediates dengue virus infection of human dendritic cells. J Exp Med.

[26] Miller JL, de Wet BJ, Martinez-Pomares L, Radcliffe CM, Dwek RA, Rudd PM (2008). The mannose receptor mediates dengue virus infection of macrophages. PLoS Pathog.

[27] Chen ST, Lin YL, Huang MT, Wu MF, Cheng SC, Lei HY (2008). CLEC5A is critical for dengue-virus-induced lethal disease. Nature.

[28] Watson AA, Lebedev AA, Hall BA, Fenton-May AE, Vagin AA, Dejnirattisai W (2011). Structural flexibility of the macrophage dengue virus receptor CLEC5A: implications for ligand binding and signaling. J Biol Chem.

[29] Modis Y, Ogata S, Clements D, Harrison SC (2004). Structure of the dengue virus envelope protein after membrane fusion. Nature.

[30] Rey FA, Heinz FX, Mandl C, Kunz C, Harrison SC (1995). The envelope glycoprotein from tick-borne encephalitis virus at 2 A resolution. Nature.

[31] Heinz FX, Allison SL (2003). Flavivirus structure and membrane fusion. Adv Virus Res.

[32] Johnson AJ, Guirakhoo F, Roehrig JT (1994). The envelope glycoproteins of dengue 1 and dengue 2 viruses grown in mosquito cells differ in their utilization of potential glycosylation sites. Virology.

[33] Lee E, Weir RC, Dalgarno L (1997). Changes in the dengue virus major envelope protein on passaging and their localization on the three-dimensional structure of the protein. Virology.

[34] Navarro-Sanchez E, Altmeyer R, Amara A, Schwartz O, Fieschi F, Virelizier JL (2003). Dendritic-cell-specific ICAM3- grabbing non-integrin is essential for the productive infection of human dendritic cells by mosquito-cell-derived dengue viruses. EMBO Rep.

[35] Clyde K, Kyle JL, Harris E (2006). Recent advances in deciphering viral and host determinants of dengue virus replication and pathogenesis. J Virol.

[36] Mackenzie JM, Jones MK, Young PR (1996). Immunolocalization of the dengue virus nonstructural glycoprotein NS1 suggests a role in viral RNA replication. Virology.

[37] Miller S, Sparacio S, Bartenschlager R (2006). Subcellular localization and membrane topology of the dengue virus type 2 Non-structural protein 4B. J Biol Chem.

[38] Salonen A, Ahola T, Kaariainen L (2005). Viral RNA replication in association with cellular membranes. Curr Top Microbiol Immunol.

[39] Lobigs M (1993). Flavivirus premembrane protein cleavage and spike heterodimer secretion require the function of the viral proteinase NS3. Proc Natl Acad Sci U S A.

[40] Yamshchikov VF, Compans RW (1993). Regulation of the late events in flavivirus protein processing and maturation. Virology.

[41] Pethel M, Falgout B, Lai CJ (1992). Mutational analysis of the octapeptide sequence motif at the NS1-NS2A cleavage junction of dengue type 4 virus. J Virol.

[42] Bazan JF, Fletterick RJ (1989). Detection of a trypsin-like serine protease domain in flaviviruses and pestiviruses. Virology.

[43] Miller S, Romero-Brey I, Bartenschlager R, Hanley KA, Weaver SC (2010). The dengue virus replication complex. Frontiers in dengue virus research.

[44] Preugschat F, Strauss JH (1991). Processing of nonstructural proteins NS4A and NS4B of dengue 2 virus in vitro and in vivo. Virology.

[45] Winkler G, Maxwell SE, Ruemmler C, Stollar V (1989). Newly synthesized dengue-2 virus nonstructural protein NS1 is a soluble protein but becomes partially hydrophobic and membrane- associated after dimerization. Virology.

[46] Libraty DH, Young PR, Pickering D, Endy TP, Kalayanarooj S, Green S (2002). High circulating levels of the dengue virus nonstructural protein NS1 early in dengue illness correlate with the development of dengue hemorrhagic fever. J Infect Dis.

[47] Avirutnan P, Fuchs A, Hauhart RE, Somnuke P, Youn S, Diamond MS (2010). Antagonism of the complement component C4 by flavivirus nonstructural protein NS1. J Exp Med.

[48] Schlesinger JJ, Brandriss MW, Walsh EE (1987). Protection of mice against dengue 2 virus encephalitis by immunization with the dengue 2 virus non-structural glycoprotein NS1. J Gen Virol.

[49] Lin CF, Wan SW, Cheng HJ, Lei HY, Lin YS (2006). Autoimmune pathogenesis in dengue virus infection. Viral Immunol.

[50] Stadler K, Allison SL, Schalich J, Heinz FX (1997). Proteolytic activation of tick-borne encephalitis virus by furin. J Virol.

[51] Guirakhoo F, Heinz FX, Mandl CW, Holzmann H, Kunz C (1991). Fusion activity of flaviviruses: comparison of mature and immature (prM-containing) tick-borne encephalitis virions. J Gen Virol.

[52] Guirakhoo F, Bolin RA, Roehrig JT (1992). The Murray Valley encephalitis virus prM protein confers acid resistance to virus particles and alters the expression of epitopes within the R2 domain of E glycoprotein. Virology.

[53] Zhang Y, Corver J, Chipman PR, Zhang W, Pletnev SV, Sedlak D (2003). Structures of immature flavivirus particles. EMBO J.

[54] Rigau-Perez JG, Clark GG, Gubler DJ, Reiter P, Sanders EJ, Vorndam AV (1998). Dengue and dengue haemorrhagic fever. Lancet.

[55] WHO (1999). Prevention and control of dengue and dengue haemorrhagic fever. Comprehensive Guidelines.

[56] Schwartz E, Mileguir F, Grossman Z, Mendelson E (2000). Evaluation of ELISA-based sero-diagnosis of dengue fever in travelers. J Clin Virol.

[57] Wang WK, Chen HL, Yang CF, Hsieh SC, Juan CC, Chang SM (2006). Slower rates of clearance of viral load and virus-containing immune complexes in patients with dengue hemorrhagic fever. Clin Infect Dis.

[58] Hang VT, Nguyet NM, Trung DT, Tricou V, Yoksan S, Dung NM (2009). Diagnostic accuracy of NS1 ELISA and lateral flow rapid tests for dengue sensitivity, specificity and relationship to viraemia and antibody responses. PLoS Negl Trop Dis.

[59] Vaughn DW, Green S, Kalayanarooj S, Innis BL, Nimmannitya S, Suntayakorn S (2000). Dengue viremia titer, antibody response pattern, and virus serotype correlate with disease severity. J Infect Dis.

[60] Young PR, Hilditch PA, Bletchly C, Halloran W (2000). An antigen capture enzyme-linked immunosorbent assay reveals high levels of the dengue virus protein NS1 in the sera of infected patients. J Clin Microbiol.

[61] Alcon S, Talarmin A, Debruyne M, Falconar A, Deubel V, Flamand M (2002). Enzyme-linked immunosorbent assay specific to dengue virus type 1 nonstructural protein NS1 reveals circulation of the antigen in the blood during the acute phase of disease in patients experiencing primary or secondary infections. J Clin Microbiol.

[62] Lapphra K, Sangcharaswichai A, Chokephaibulkit K, Tiengrim S, Piriyakarnsakul W, Chakorn T (2008). Evaluation of an NS1 antigen detection for diagnosis of acute dengue infection in patients with acute febrile illness. Diagn Microbiol Infect Dis.

[63] Schilling S, Ludolfs D, Van An L, Schmitz H (2004). Laboratory diagnosis of primary and secondary dengue infection. J Clin Virol.

[64] Dussart P, Petit L, Labeau B, Bremand L, Leduc A, Moua D (2008). Evaluation of two new commercial tests for the diagnosis of acute dengue virus infection using NS1 antigen detection in human serum. PLoS Negl Trop Dis.

[65] Phuong HL, Thai KT, Nga TT, Giao PT, Hung le Q, Binh TQ (2009). Detection of dengue nonstructural 1 (NS1) protein in Vietnamese patients with fever. Diagn Microbiol Infect Dis.

[66] Duong V, Ly S, Lorn Try P, Tuiskunen A, Ong S, Chroeung N (2011). Clinical and virological factors influencing the performance of a NS1 antigen-capture assay and potential use as a marker of dengue disease severity. PLoS Negl Trop Dis.

[67] Gibbons RV, Kalanarooj S, Jarman RG, Nisalak A, Vaughn DW, Endy TP (2007). Analysis of repeat hospital admissions for dengue to estimate the frequency of third or fourth dengue infections resulting in admissions and dengue hemorrhagic fever, and serotype sequences. Am J Trop Med Hyg.

[68] Raviprakash K, Apt D, Brinkman A, Skinner C, Yang S, Dawes G (2006). A chimeric tetravalent dengue DNA vaccine elicits neutralizing antibody to all four virus serotypes in rhesus macaques. Virology.

[69] Konishi E, Kosugi S, Imoto J (2006). Dengue tetravalent DNA vaccine inducing neutralizing antibody and anamnestic responses to four serotypes in mice. Vaccine.

[70] Wisseman CL, Sweet BH, Rosenzweig EC, Eylar OR (1963). Attenuated living type I dengue vaccines. Am J Trop Med Hyg.

[71] Guirakhoo F, Kitchener S, Morrison D, Forrat R, McCarthy K, Nichols R (2006). Live attenuated chimeric yellow fever dengue type 2 (ChimeriVax-DEN2) vaccine: Phase I clinical trial for safety and immunogenicity: effect of yellow fever preimmunity in induction of cross neutralizing antibody responses to all 4 dengue serotypes. Hum Vaccin.

[72] Kanesa-Thasan N, Edelman R, Tacket CO, Wasserman SS, Vaughn DW, Coster TS (2003). Phase 1 studies of Walter Reed Army Institute of Research candidate attenuated dengue vaccines: selection of safe and immunogenic monovalent vaccines. Am J Trop Med Hyg.

[73] Sabchareon A, Lang J, Chanthavanich P, Yoksan S, Forrat R, Attanath P (2002). Safety and immunogenicity of tetravalent live-attenuated dengue vaccines in Thai adult volunteers: role of serotype concentration, ratio, and multiple doses. Am J Trop Med Hyg.

[74] Edelman R, Tacket CO, Wasserman SS, Vaughn DW, Eckels KH, Dubois DR (1994). A live attenuated dengue-1 vaccine candidate (45AZ5) passaged in primary dog kidney cell culture is attenuated and immunogenic for humans. J Infect Dis.

[75] Sun W, Edelman R, Kanesa-Thasan N, Eckels KH, Putnak JR, King AD (2003). Vaccination of human volunteers with monovalent and tetravalent live-attenuated dengue vaccine candidates. Am J Trop Med Hyg.

[76] Men R, Bray M, Clark D, Chanock RM, Lai CJ (1996). Dengue type 4 virus mutants containing deletions in the 3’ noncoding region of the RNA genome: analysis of growth restriction in cell culture and altered viremia pattern and immunogenicity in rhesus monkeys. J Virol.

[77] Guirakhoo F, Arroyo J, Pugachev KV, Miller C, Zhang ZX, Weltzin R (2001). Construction, safety, and immunogenicity in nonhuman primates of a chimeric yellow fever-dengue virus tetravalent vaccine. J Virol.

[78] Durbin AP, Whitehead SS (2011). Next-generation dengue vaccines: novel strategies currently under development. Viruses.

[79] Gubler DJ (1998). The global pandemic of dengue/dengue haemorrhagic fever: current status and prospects for the future. Ann Acad Med Singap.

[80] Halstead SB, Deen J (2002). The future of dengue vaccines. Lancet.

[81] Wills BA, Nguyen MD, Ha TL, Dong TH, Tran TN, Le TT (2005). Comparison of three fluid solutions for resuscitation in dengue shock syndrome. N Engl J Med.

[82] LaBauve ME, Kuhn RJ, Hanley KA, Weaver SC (2010). Novel therapeutic approaches for dengue disease. Frontiers in dengue virus research.

[83] Gubler DJ (1998). Dengue and dengue hemorrhagic fever. Clin Microbiol Rev.

[84] Guzman MG, Kouri G, Bravo J, Valdes L, Vazquez S, Halstead SB (2002). Effect of age on outcome of secondary dengue 2 infections. Int J Infect Dis.

[85] Guzman MG, Kouri G, Valdes L, Bravo J, Vazquez S, Halstead SB (2002). Enhanced severity of secondary dengue-2 infections: death rates in 1981 and 1997 Cuban outbreaks. Rev Panam Salud Publica.

[86] Halstead SB, Streit TG, Lafontant JG, Putvatana R, Russell K, Sun W (2001). Haiti: absence of dengue hemorrhagic fever despite hyperendemic dengue virus transmission. Am J Trop Med Hyg.

[87] Balmaseda A, Hammond SN, Perez L, Tellez Y, Saborio SI, Mercado JC (2006). Serotype-specific differences in clinical manifestations of dengue. Am J Trop Med Hyg.

[88] Messer WB, Gubler DJ, Harris E, Sivananthan K, de Silva AM (2003). Emergence and global spread of a dengue serotype 3, subtype III virus. Emerg Infect Dis.

[89] Rico-Hesse R, Harrison LM, Salas RA, Tovar D, Nisalak A, Ramos C (1997). Origins of dengue type 2 viruses associated with increased pathogenicity in the Americas. Virology.

[90] Burke DS, Nisalak A, Johnson DE, Scott RM (1988). A prospective study of dengue infections in Bangkok. Am J Trop Med Hyg.

[91] Halstead SB, Nimmannitya S, Cohen SN (1970). Observations related to pathogenesis of dengue hemorrhagic fever. IV. Relation of disease severity to antibody response and virus recovered. Yale J Biol Med.

[92] Sangkawibha N, Rojanasuphot S, Ahandrik S, Viriyapongse S, Jatanasen S, Salitul V (1984). Risk factors in dengue shock syndrome: a prospective epidemiologic study in Rayong, Thailand. I. The outbreak 1980. Am J Epidemiol.

[93] Thein S, Aung MM, Shwe TN, Aye M, Zaw A, Aye K (1997). Risk factors in dengue shock syndrome. Am J Trop Med Hyg.

[94] Guzman MG, Sierra B, Kouri G, Farrar J, Simmons C, Hanley KA, Weaver SC (2010). Host and virus determinants of susceptibility and dengue disease severity. Frontiers in dengue virus research.

[95] Bravo JR, Guzman MG, Kouri GP (1987). Why dengue haemorrhagic fever in Cuba? 1. Individual risk factors for dengue haemorrhagic fever/dengue shock syndrome (DHF/DSS). Trans R Soc Trop Med Hyg.

[96] Ferguson NM, Donnelly CA, Anderson RM (1999). Transmission dynamics and epidemiology of dengue: insights from age-stratified sero-prevalence surveys. Philos Trans R Soc Lond B Biol Sci.

[97] Dejnirattisai W, Jumnainsong A, Onsirisakul N, Fitton P, Vasanawathana S, Limpitikul W (2010). Cross-reacting antibodies enhance dengue virus infection in humans. Science.

[98] Rodenhuis-Zybert IA, van der Schaar HM, da Silva Voorham JM, van der Ende-Metselaar H, Lei HY, Wilschut J (2010). Immature dengue virus: a veiled pathogen?. PLoS Pathog.

[99] Guzman MG, Alvarez M, Rodriguez-Roche R, Bernardo L, Montes T, Vazquez S (2007). Neutralizing antibodies after infection with dengue 1 virus. Emerg Infect Dis.

[100] Chau TN, Hieu NT, Anders KL, Wolbers M, Lien le B, Hieu LT (2009). Dengue virus infections and maternal antibody decay in a prospective birth cohort study of Vietnamese infants. J Infect Dis.

[101] Pengsaa K, Luxemburger C, Sabchareon A, Limkittikul K, Yoksan S, Chambonneau L (2006). Dengue virus infections in the first 2 years of life and the kinetics of transplacentally transferred dengue neutralizing antibodies in thai children. J Infect Dis.

[102] Azeredo EL, Zagne SM, Santiago MA, Gouvea AS, Santana AA, Neves-Souza PC (2001). Characterisation of lymphocyte response and cytokine patterns in patients with dengue fever. Immunobiology.

[103] Chakravarti A, Kumaria R (2006). Circulating levels of tumour necrosis factor-alpha & interferon-gamma in patients with dengue & dengue haemorrhagic fever during an outbreak. Indian J Med Res.

[104] Nguyen TH, Lei HY, Nguyen TL, Lin YS, Huang KJ, Le BL (2004). Dengue hemorrhagic fever in infants: a study of clinical and cytokine profiles. J Infect Dis.

[105] Mustafa AS, Elbishbishi EA, Agarwal R, Chaturvedi UC (2001). Elevated levels of interleukin-13 and IL-18 in patients with dengue hemorrhagic fever. FEMS Immunol Med Microbiol.

[106] Perez AB, Garcia G, Sierra B, Alvarez M, Vazquez S, Cabrera MV (2004). IL-10 levels in dengue patients: some findings from the exceptional epidemiological conditions in Cuba. J Med Virol.

[107] Pinto LM, Oliveira SA, Braga EL, Nogueira RM, Kubelka CF (1999). Increased pro-inflammatory cytokines (TNF-alpha and IL-6) and anti-inflammatory compounds (sTNFRp55 and sTNFR p75) in Brazilian patients during exanthematic dengue fever. Mem Inst Oswaldo Cruz.

[108] Yang KD, Wang CL, Shaio MF (1995). Production of cytokines and platelet activating factor in secondary dengue virus infections. J Infect Dis.

[109] Libraty DH, Endy TP, Houng HS, Green S, Kalayanarooj S, Suntayakorn S (2002). Differing influences of virus burden and immune activation on disease severity in secondary dengue-3 virus infections. J Infect Dis.

[110] Wang WK, Chao DY, Kao CL, Wu HC, Liu YC, Li CM (2003). High levels of plasma dengue viral load during defervescence in patients with dengue hemorrhagic fever: implications for pathogenesis. Virology.

[111] Vaughn DW, Green S, Kalayanarooj S, Innis BL, Nimmannitya S, Suntayakorn S (1997). Dengue in the early febrile phase: viremia and antibody responses. J Infect Dis.

[112] Laoprasopwattana K, Libraty DH, Endy TP, Nisalak A, Chunsuttiwat S, Vaughn DW (2005). Dengue virus (DV) enhancing antibody activity in preillness plasma does not predict subsequent disease severity or viremia in secondary DV infection. J Infect Dis.

[113] Libraty DH, Acosta LP, Tallo V, Segubre-Mercado E, Bautista A, Potts JA (2009). A prospective nested case-control study of dengue in infants: rethinking and refining the antibody-dependent enhancement dengue hemorrhagic fever model. PLoS Med.

[114] Endy TP, Nisalak A, Chunsuttitwat S, Vaughn DW, Green S, Ennis FA (2004). Relationship of preexisting dengue virus (DV) neutralizing antibody levels to viremia and severity of disease in a prospective cohort study of DV infection in Thailand. J Infect Dis.

[115] Halstead SB (2007). Dengue. Lancet.

[116] Alvarez M, Rodriguez-Roche R, Bernardo L, Vazquez S, Morier L, Gonzalez D (2006). Dengue hemorrhagic fever caused by sequential dengue 1–3 virus infections over a long time interval: havana epidemic, 2001–2002. Am J Trop Med Hyg.

[117] Westaway EG, Blok J, Gubler DJ, Kuno G (1997). Taxonomy an evolutionary relationships of flaviviruses. Dengue an dengue hemorrhagic fever.

[118] Barnes WJ, Rosen L (1974). Fatal hemorrhagic disease and shock associated with primary dengue infection on a Pacific island. Am J Trop Med Hyg.

[119] Rosen L (1977). The emperor's new lothes revisited, or reflections on the pathogenesis of dengue hemorrhagic fever. Am J Trop Med Hyg.

[120] Gubler DJ, Suharyono W, Lubis I, Eram S, Gunarso S (1981). Epidemic dengue 3 in central Java, associated with low viremia in man. Am J Trop Med Hyg.

[121] Anderson JR, Rico-Hesse R (2006). Aedes aegypti vectorial capacity is determined by the infecting genotype of dengue virus. Am J Trop Med Hyg.

[122] Rico-Hesse R (1990). Molecular evolution and distribution of dengue viruses type 1 and 2 in nature. Virology.

[123] Messer WB, Vitarana UT, Sivananthan K, Elvtigala J, Preethimala LD, Ramesh R (2002). Epidemiology of dengue in Sri Lanka before and after the emergence of epidemic dengue hemorrhagic fever. Am J Trop Med Hyg.

[124] Kanakaratne N, Wahala WM, Messer WB, Tissera HA, Shahani A, Abeysinghe N (2009). Severe dengue epidemics in Sri Lanka, 2003–2006. Emerg Infect Dis.

[125] Hanley KA, Nelson JT, Schirtzinger EE, Whitehead SS, Hanson CT (2008). Superior infectivity for mosquito vectors contributes to competitive displacement among strains of dengue virus. BMC Ecol.

[126] Vasilakis N, Shell EJ, Fokam EB, Mason PW, Hanley KA, Estes DM (2007). Potential of ancestral sylvatic dengue-2 viruses to re-emerge. Virology.

[127] Leitmeyer KC, Vaughn DW, Watts DM, Salas R, Villalobos I, de C (1999). Dengue virus structural differences that correlate with pathogenesis. J Virol.

[128] Kochel TJ, Watts DM, Gozalo AS, Ewing DF, Porter KR, Russell KL (2005). Cross-serotype neutralization of dengue virus in Aotus nancymae monkeys. J Infect Dis.

[129] Bhamarapravati N (1989). Hemostatic defects in dengue hemorrhagic fever. Rev Infect Dis.

[130] Hotta S (1969). Propagation of dengue virus in tissue culture. Acta Trop.

[131] Bhamarapravati N, Toochinda P, Boonyapaknavik V (1967). Pathology of Thailand haemorrhagic fever: a study of 100 autopsy cases. Am J Trop Med Parasitol.

[132] Ho LJ, Wang JJ, Shaio MF, Kao CL, Chang DM, Han SW (2001). Infection of human dendritic cells by dengue virus causes cell maturation and cytokine production. J Immunol.

[133] Libraty DH, Pichyangkul S, Ajariyakhajorn C, Endy TP, Ennis FA (2001). Human dendritic cells are activated by dengue virus infection: enhancement by gamma interferon and implications for disease pathogenesis. J Virol.

[134] Marovich M, Grouard-Vogel G, Louder M, Eller M, Sun W, Wu SJ (2001). Human dendritic cells as targets of dengue virus infection. J Investig Dermatol Symp Proc.

[135] Wu SJ, Grouard-Vogel G, Sun W, Mascola JR, Brachtel E, Putvatana R (2000). Human skin Langerhans cells are targets of dengue virus infection. Nat Med.

[136] Johnston LJ, Halliday GM, King NJ (2000). Langerhans cells migrate to local lymph nodes following cutaneous infection with an arbovirus. J Invest Dermatol.

[137] Boonpucknavig S, Boonpucknavig V, Bhamarapravati N, Nimmannitya S (1979). Immunofluorescence study of skin rash in patients with dengue hemorrhagic fever. Arch Pathol Lab Med.

[138] Sahaphong S, Riengrojpitak S, Bhamarapravati N, Chirachariyavej T (1980). Electron microscopic study of the vascular endothelial cell in dengue hemorrhagic fever. Southeast Asian J Trop Med Public Health.

[139] Scott RM, Nisalak A, Cheamudon U, Seridhoranakul S, Nimmannitya S (1980). Isolation of dengue viruses from peripheral blood leukocytes of patients with hemorrhagic fever. J Infect Dis.

[140] Jessie K, Fong MY, Devi S, Lam SK, Wong KT (2004). Localization of dengue virus in naturally infected human tissues, by immunohistochemistry and in situ hybridization. J Infect Dis.

[141] Diamond MS, Shrestha B, Marri A, Mahan D, Engle M (2003). B cells and antibody play critical roles in the immediate defense of disseminated infection by West Nile encephalitis virus. J Virol.

[142] Solomon T, Vaughn DW (2002). Pathogenesis and clinical features of Japanese encephalitis and West Nile virus infections. Curr Top Microbiol Immunol.

[143] Xiao SY, Guzman H, Zhang H, Travassos da Rosa AP, Tesh RB (2001). West Nile virus infection in the golden hamster (Mesocricetus auratus): a model for West Nile encephalitis. Emerg Infect Dis.

[144] Xiao SY, Zhang H, Guzman H, Tesh RB (2001). Experimental yellow fever virus infection in the golden hamster (Mesocricetus auratus). II. Pathology. J Infect Dis.

[145] Durbin AP, Vargas MJ, Wanionek K, Hammond SN, Gordon A, Rocha C (2008). Phenotyping of peripheral blood mononuclear cells during acute dengue illness demonstrates infection and increased activation of monocytes in severe cases compared to classic dengue fever. Virology.

[146] Marchette NJ, Halstead SB, Falkler WA, Stenhouse A, Nash D (1973). Studies on the pathogenesis of dengue infection in monkeys. 3. Sequential distribution of virus in primary and heterologous infections. J Infect Dis.

[147] Bhoopat L, Bhamarapravati N, Attasiri C, Yoksarn S, Chaiwun B, Khunamornpong S (1996). Immunohistochemical characterization of a new monoclonal antibody reactive with dengue virus-infected cells in frozen tissue using immunoperoxidase technique. Asian Pac J Allergy Immunol.

[148] Couvelard A, Marianneau P, Bedel C, Drouet MT, Vachon F, Henin D (1999). Report of a fatal case of dengue infection with hepatitis: demonstration of dengue antigens in hepatocytes and liver apoptosis. Hum Pathol.

[149] Huerre MR, Lan NT, Marianneau P, Hue NB, Khun H, Hung NT (2001). Liver histopathology and biological correlates in five cases of fatal dengue fever in Vietnamese children. Virchows Arch.

[150] Miagostovich MP, Ramos RG, Nicol AF, Nogueira RM, Cuzzi-Maya T, Oliveira AV (1997). Retrospective study on dengue fatal cases. Clin Neuropathol.

[151] Hall WC, Crowell TP, Watts DM, Barros VL, Kruger H, Pinheiro F (1991). Demonstration of yellow fever and dengue antigens in formalin-fixed paraffin-embedded human liver by immunohistochemical analysis. Am J Trop Med Hyg.

[152] Ramos C, Sanchez G, Pando RH, Baquera J, Hernandez D, Mota J (1998). Dengue virus in the brain of a fatal case of hemorrhagic dengue fever. J Neurovirol.

[153] Andrews BS, Theofilopoulos AN, Peters CJ, Loskutoff DJ, Brandt WE, Dixon FJ (1978). Replication of dengue and junin viruses in cultured rabbit and human endothelial cells. Infect Immun.

[154] Balsitis SJ, Coloma J, Castro G, Alava A, Flores D, McKerrow JH (2009). Tropism of dengue virus in mice and humans defined by viral nonstructural protein 3-specific immunostaining. Am J Trop Med Hyg.

[155] Wang L, Chen RF, Liu JW, Lee IK, Lee CP, Kuo HC (2011). DC-SIGN (CD209) Promoter -336 A/G polymorphism is associated with dengue hemorrhagic fever and correlated to DC-SIGN expression and immune augmentation. PLoS Negl Trop Dis.

[156] Avirutnan P, Zhang L, Punyadee N, Manuyakorn A, Puttikhunt C, Kasinrerk W (2007). Secreted NS1 of dengue virus attaches to the surface of cells via interactions with heparan sulfate and chondroitin sulfate E. PLoS Pathog.

[157] Whitehorn J, Simmons CP (2011). The pathogenesis of dengue. Vaccine.

[158] Valdes K, Alvarez M, Pupo M, Vazquez S, Rodriguez R, Guzman MG (2000). Human dengue antibodies against structural and nonstructural proteins. Clin Diagn Lab Immunol.

[159] Churdboonchart V, Bhamarapravati N, Peampramprecha S, Sirinavin S (1991). Antibodies against dengue viral proteins in primary and secondary dengue hemorrhagic fever. Am J Trop Med Hyg.

[160] Roehrig JT, Bolin RA, Kelly RG (1998). Monoclonal antibody mapping of the envelope glycoprotein of the dengue 2 virus, Jamaica. Virology.

[161] Sukupolvi-Petty S, Austin SK, Engle M, Brien JD, Dowd KA, Williams KL (2010). Structure and function analysis of therapeutic monoclonal antibodies against dengue virus type 2. J Virol.

[162] Lok SM, Kostyuchenko V, Nybakken GE, Holdaway HA, Battisti AJ, Sukupolvi-Petty S (2008). Binding of a neutralizing antibody to dengue virus alters the arrangement of surface glycoproteins. Nat Struct Mol Biol.

[163] Kaufmann B, Nybakken GE, Chipman PR, Zhang W, Diamond MS, Fremont DH (2006). West Nile virus in complex with the Fab fragment of a neutralizing monoclonal antibody. Proc Natl Acad Sci U S A.

[164] Cherrier MV, Kaufmann B, Nybakken GE, Lok SM, Warren JT, Chen BR (2009). Structural basis for the preferential recognition of immature flaviviruses by a fusion-loop antibody. EMBO J.

[165] Lai CY, Tsai WY, Lin SR, Kao CL, Hu HP, King CC (2008). Antibodies to envelope glycoprotein of dengue virus during the natural course of infection are predominantly cross-reactive and recognize epitopes containing highly conserved residues at the fusion loop of domain II. J Virol.

[166] Lin B, Parrish CR, Murray JM, Wright PJ (1994). Localization of a neutralizing epitope on the envelope protein of dengue virus type 2. Virology.

[167] Lok SM, Ng ML, Aaskov J (2001). Amino acid and phenotypic changes in dengue 2 virus associated with escape from neutralisation by IgM antibody. J Med Virol.

[168] Wang WK, Lin SR, Lee CM, King CC, Chang SC (2002). Dengue type 3 virus in plasma is a population of closely related genomes: quasispecies. J Virol.

[169] Wang WK, Sung TL, Lee CN, Lin TY, King CC (2002). Sequence diversity of the capsid gene and the nonstructural gene NS2B of dengue-3 virus in vivo. Virology.

[170] Mehlhop E, Ansarah-Sobrinho C, Johnson S, Engle M, Fremont DH, Pierson TC (2007). Complement protein C1q inhibits antibody-dependent enhancement of flavivirus infection in an IgG subclass-specific manner. Cell Host Microbe.

[171] Bokisch VA, Top FH, Russell PK, Dixon FJ, Muller- Eberhard HJ (1973). The potential pathogenic role of complement in dengue hemorrhagic shock syndrome. N Engl J Med.

[172] Malasit P (1987). Complement and dengue haemorrhagic fever/shock syndrome. Southeast Asian J Trop Med Public Health.

[173] Mentor NA, Kurane I (1997). Dengue virus infection of human T lymphocytes. Acta Virol.

[174] Gagnon SJ, Ennis FA, Rothman AL (1999). Bystander target cell lysis and cytokine production by dengue virus-specific human CD4(+) cytotoxic T-lymphocyte clones. J Virol.

[175] Mangada MM, Ennis FA, Rothman AL (2004). Quantitation of dengue virus specific CD4+ T cells by intracellular cytokine staining. J Immunol Methods.

[176] Bashyam HS, Green S, Rothman AL (2006). Dengue virus-reactive CD8+ T cells display quantitative and qualitative differences in their response to variant epitopes of heterologous viral serotypes. J Immunol.

[177] Imrie A, Meeks J, Gurary A, Sukhbataar M, Kitsutani P, Effler P (2007). Differential functional avidity of dengue virus-specific T-cell clones for variant peptides representing heterologous and previously encountered serotypes. J Virol.

[178] Dong T, Moran E, Vinh Chau N, Simmons C, Luhn K, Peng Y (2007). High pro-inflammatory cytokine secretion and loss of high avidity cross-reactive cytotoxic T-cells during the course of secondary dengue virus infection. PLoS One.

[179] Duangchinda T, Dejnirattisai W, Vasanawathana S, Limpitikul W, Tangthawornchaikul N, Malasit P (2010). Immunodominant T-cell responses to dengue virus NS3 are associated with DHF. Proc Natl Acad Sci U S A.

[180] An J, Zhou DS, Zhang JL, Morida H, Wang JL, Yasui K (2004). Dengue-specific CD8+ T cells have both protective and pathogenic roles in dengue virus infection. Immunol Lett.

[181] Luhn K, Simmons CP, Moran E, Dung NT, Chau TN, Quyen NT (2007). Increased frequencies of CD4+ CD25(high) regulatory T cells in acute dengue infection. J Exp Med.

[182] Kurane I, Innis BL, Nimmannitya S, Nisalak A, Rothman AL, Livingston PG (1990). Human immune responses to dengue viruses. Southeast Asian J Trop Med Public Health.

[183] Rothman AL, Ennis FA (1999). Immunopathogenesis of dengue hemorrhagic fever. Virology.

[184] Zivna I, Green S, Vaughn DW, Kalayanarooj S, Stephens HA, Chandanayingyong D (2002). T cell responses to an HLA-B*07-restricted epitope on the dengue NS3 protein correlate with disease severity. J Immunol.

[185] Mongkolsapaya J, Dejnirattisai W, Xu XN, Vasanawathana S, Tangthawornchaikul N, Chairunsri A (2003). Original antigenic sin and apoptosis in the pathogenesis of dengue hemorrhagic fever. Nat Med.

[186] Mangada MM, Rothman AL (2005). Altered cytokine responses of dengue-specific CD4+ T cells to heterologous serotypes. J Immunol.

[187] Valdes L, Guzman MG, Kouri G, Delgado J, Carbonell I, Cabrera MV (1999). Epidemiology of dengue and hemorrhagic dengue in Santiago, Cuba 1997. Rev Panam Salud Publica.

[188] Halstead SB, Rojanasuphot S, Sangkawibha N (1983). Original antigenic sin in dengue. Am J Trop Med Hyg.

[189] Guzman MG, Alvarez M, Rodriguez R, Rosario D, Vazquez S, Vald S (1999). Fatal dengue hemorrhagic fever in Cuba, 1997. Int J Infect Dis.

[190] Pelaez O, Guzman MG, Kouri G, Perez R, San Martin JL, Vazquez S (2004). Dengue 3 epidemic, Havana, 2001. Emerg Infect Dis.

[191] Akira S, Takeda K (2004). Toll-like receptor signalling. Nat Rev Immunol.

[192] Bowie AG, Haga IR (2005). The role of toll-like receptors in the host response to viruses. Mol Immunol.

[193] Meylan E, Tschopp J (2006). Toll-like receptors and RNA helicases: two parallel ways to trigger antiviral responses. Mol Cell.

[194] Severa M, Fitzgerald KA (2007). TLR-mediated activation of type I IFN during antiviral immune responses: fighting the battle to win the war. Curr Top Microbiol Immunol.

[195] Anderson R (2003). Manipulation of cell surface macromolecules by flaviviruses. Adv Virus Res.

[196] Carr JM, Hocking H, Bunting K, Wright PJ, Davidson A, Gamble J (2003). Supernatants from dengue virus type-2 infected macrophages induce permeability changes in endothelial cell monolayers. J Med Virol.

[197] Espina LM, Valero NJ, Hernandez JM, Mosquera JA (2003). Increased apoptosis and expression of tumor necrosis factoralpha caused by infection of cultured human monocytes with dengue virus. Am J Trop Med Hyg.

[198] Charnsilpa W, Takhampunya R, Endy TP, Mammen MP, Libraty DH, Ubol S (2005). Nitric oxide radical suppresses replication of wild-type dengue 2 viruses in vitro. J Med Virol.

[199] Neves-Souza PC, Azeredo EL, Zagne SM, Valls-de-Souza R, Reis SR, Cerqueira DI (2005). Inducible nitric oxide synthase (iNOS) expression in monocytes during acute Dengue Fever in patients and during in vitro infection. BMC Infect Dis.

[200] Borish LC, Steinke JW (2003). 2. Cytokines and chemokines. J Allergy Clin Immunol.

[201] Basu A, Chaturvedi UC (2008). Vascular endothelium: the battlefield of dengue viruses. FEMS Immunol Med Microbiol.

[202] Green S, Vaughn DW, Kalayanarooj S, Nimmannitya S, Suntayakorn S, Nisalak A (1999). Elevated plasma interleukin- 10 levels in acute dengue correlate with disease severity. J Med Virol.

[203] Kurane I, Innis BL, Nimmannitya S, Nisalak A, Meager A, Janus J (1991). Activation of T lymphocytes in dengue virus infections. High levels of soluble interleukin 2 receptor, soluble CD4, soluble CD8, interleukin 2, and interferon-gamma in sera of children with dengue. J Clin Invest.

[204] Hober D, Poli L, Roblin B, Gestas P, Chungue E, Granic G (1993). Serum levels of tumor necrosis factor-alpha (TNF-alpha), interleukin-6 (IL-6), and interleukin-1 beta (IL-1 beta) in dengue-infected patients. Am J Trop Med Hyg.

[205] Iyngkaran N, Yadav M, Sinniah M (1995). Augmented inflammatory cytokines in primary dengue infection progressing to shock. Singap Med J.

[206] Juffrie M, Meer GM, Hack CE, Haasnoot K, Sutaryo AJ, Veerman (2001). Inflammatory mediators in dengue virus infection in children: interleukin-6 and its relation to C-reactive protein and secretory phospholipase A2. Am J Trop Med Hyg.

[207] Talavera D, Castillo AM, Dominguez MC, Gutierrez AE, Meza I (2004). IL8 release, tight junction and cytoskeleton dynamic reorganization conducive to permeability increase are induced by dengue virus infection of microvascular endothelial monolayers. J Gen Virol.

[208] Hober D, Shen L, Benyoucef S, De Groote D, Deubel V, Wattre P (1996). Enhanced TNF alpha production by monocytic-like cells exposed to dengue virus antigens. Immunol Lett.

[209] Bethell DB, Flobbe K, Cao XT, Day NP, Pham TP, Buurman WA (1998). Pathophysiologic and prognostic role of cytokines in dengue hemorrhagic fever. J Infect Dis.

[210] Sierra B, Perez AB, Vogt K, Garcia G, Schmolke K, Aguirre E (2010). MCP-1 and MIP-1alpha expression in a model resembling early immune response to dengue. Cytokine.

[211] Mosmann TR, Sad S (1996). The expanding universe of T-cell subsets: Th1, Th2 and more. Immunol Today.

[212] Chen HC, Hofman FM, Kung JT, Lin YD, Wu-Hsieh BA (2007). Both virus and tumor necrosis factor alpha are critical for endothelium damage in a mouse model of dengue virus-induced hemorrhage. J Virol.

[213] Lei HY, Yeh TM, Liu HS, Lin YS, Chen SH, Liu CC (2001). Immunopathogenesis of dengue virus infection. J Biomed Sci.

[214] Martina BE, Koraka P, Osterhaus AD (2009). Dengue virus pathogenesis: an integrated view. Clin Microbiol Rev.

[215] Kallmann BA, Hummel V, Lindenlaub T, Ruprecht K, Toyka KV, Rieckmann P (2000). Cytokine-induced modulation of cellular adhesion to human cerebral endothelial cells is mediated by soluble vascular cell adhesion molecule-1. Brain.

[216] Madan B, Singh I, Kumar A, Prasad AK, Raj HG, Parmar VS (2002). Xanthones as inhibitors of microsomal lipid peroxidation and TNF-alpha induced ICAM-1 expression on human umbilical vein endothelial cells (HUVECs). Bioorg Med Chem.

[217] Dagia NM, Goetz DJ (2003). A proteasome inhibitor reduces concurrent, sequential, and long-term IL-1 beta- and TNF-alpha-induced ECAM expression and adhesion. Am J Physiol Cell Physiol.

[218] Javaid K, Rahman A, Anwar KN, Frey RS, Minshall RD, Malik AB (2003). Tumor necrosis factor-alpha induces early-onset endothelial adhesivity by protein kinase Czeta-dependent activation of intercellular adhesion molecule-1. Circ Res.

[219] Anderson R, Wang S, Osiowy C, Issekutz AC (1997). Activation of endothelial cells via antibody-enhanced dengue virus infection of peripheral blood monocytes. J Virol.

[220] Weaver KA, H.a.S.C (2010). Frontiers in dengue virus research. Chapter 6.

[221] Tuiskunen A, Monteil V, Plumet S, Boubis L, Wahlstrom M, Duong V (2011). Phenotypic and genotypic characterization of dengue virus isolates differentiates dengue fever and dengue hemorrhagic fever from dengue shock syndrome. Arch Virol.

[222] Barreto DF, Takiya CM, Schatzmayr HG, Nogueira RM, Farias-Filho Jda C, Barth OM (2007). Histopathological and ultrastructural aspects of mice lungs experimentally infected with dengue virus serotype 2. Mem Inst Oswaldo Cruz.

[223] Paes MV, Pinhao AT, Barreto DF, Costa SM, Oliveira MP, Nogueira AC (2005). Liver injury and viremia in mice infected with dengue-2 virus. Virology.

[224] Huang KJ, Li SY, Chen SC, Liu HS, Lin YS, Yeh TM (2000). Manifestation of thrombocytopenia in dengue-2-virus-infected mice. J Gen Virol.

[225] Tuiskunen A, Wahlstrom M, Bergstrom J, Buchy P, Leparc-Goffart I, Lundkvist A (2011). Phenotypic characterization of patient dengue virus isolates in BALB/c mice differentiates dengue fever and dengue hemorrhagic fever from dengue shock syndrome. Virol J.

[226] Atrasheuskaya A, Petzelbauer P, Fredeking TM, Ignatyev G (2003). Anti-TNF antibody treatment reduces mortality in experimental dengue virus infection. FEMS Immunol Med Microbiol.

[227] Zulueta A, Martin J, Hermida L, Alvarez M, Valdes I, Prado I (2006). Amino acid changes in the recombinant dengue 3 envelope domain III determine its antigenicity and immunogenicity in mice. Virus Res.

[228] An J, Kimura-Kuroda J, Hirabayashi Y, Yasui K (1999). Development of a novel mouse model for dengue virus infection. Virology.

[229] Lin YL, Liao CL, Chen LK, Yeh CT, Liu CI, Ma SH (1998). Study of dengue virus infection in SCID mice engrafted with human K562 cells. J Virol.

[230] Blaney JE, Johnson DH, Manipon GG, Firestone CY, Hanson CT, Murphy BR (2002). Genetic basis of attenuation of dengue virus type 4 small plaque mutants with restricted replication in suckling mice and in SCID mice transplanted with human liver cells. Virology.

[231] Bente DA, Melkus MW, Garcia JV, Rico-Hesse R (2005). Dengue fever in humanized NOD/SCID mice. J Virol.

[232] Kuruvilla JG, Troyer RM, Devi S, Akkina R (2007). Dengue virus infection and immune response in humanized RAG2(-/-) gamma(c)(-/-) (RAG-hu) mice. Virology.

[233] Shultz LD, Schweitzer PA, Christianson SW, Gott B, Schweitzer IB, Tennent B (1995). Multiple defects in innate and adaptive immunologic function in NOD/LtSz-scid mice. J Immunol.

[234] Cao X, Shores EW, Hu-Li J, Anver MR, Kelsall BL, Russell SM (1995). Defective lymphoid development in mice lacking expression of the common cytokine receptor gamma chain. Immunity.

[235] DiSanto JP, Muller W, Guy-Grand D, Fischer A, Rajewsky K (1995). Lymphoid development in mice with a targeted deletion of the interleukin 2 receptor gamma chain. Proc Natl Acad Sci U S A.

[236] Ohbo K, Suda T, Hashiyama M, Mantani A, Ikebe M, Miyakawa K (1996). Modulation of hematopoiesis in mice with a truncated mutant of the interleukin-2 receptor gamma chain. Blood.

[237] Ifversen P, Borrebaeck CA (1996). SCID-hu-PBL: a model for making human antibodies?. Semin Immunol.

[238] Murphy WJ, Taub DD, Longo DL (1996). The huPBL-SCID mouse as a means to examine human immune function in vivo. Semin Immunol.

[239] Shultz LD, Lyons BL, Burzenski LM, Gott B, Chen X, Chaleff S (2005). Human lymphoid and myeloid cell development in NOD/LtSz-scid IL2R gamma null mice engrafted with mobilized human hemopoietic stem cells. J Immunol.

[240] Ishikawa F, Yasukawa M, Lyons B, Yoshida S, Miyamoto T, Yoshimoto G (2005). Development of functional human blood and immune systems in NOD/SCID/IL2 receptor {gamma} chain(null) mice. Blood.

[241] Johnson AJ, Roehrig JT (1999). New mouse model for dengue virus vaccine testing. J Virol.

[242] Shresta S, Kyle JL, Snider HM, Basavapatna M, Beatty PR, Harris E (2004). Interferon-dependent immunity is essential for resistance to primary dengue virus infection in mice, whereas T- and B-cell-dependent immunity are less critical. J Virol.

[243] Kyle JL, Beatty PR, Harris E (2007). Dengue virus infects macrophages and dendritic cells in a mouse model of infection. J Infect Dis.

[244] Huang CY, Butrapet S, Tsuchiya KR, Bhamarapravati N, Gubler DJ, Kinney RM (2003). Dengue 2 PDK-53 virus as a chimeric carrier for tetravalent dengue vaccine development. J Virol.

[245] Lee E, Wright PJ, Davidson A, Lobigs M (2006). Virulence attenuation of dengue virus due to augmented glycosaminoglycanbinding affinity and restriction in extraneural dissemination. J Gen Virol.

[246] Schul W, Liu W, Xu HY, Flamand M, Vasudevan SG (2007). A dengue fever viremia model in mice shows reduction in viral replication and suppression of the inflammatory response after treatment with antiviral drugs. J Infect Dis.

[247] Shresta S, Sharar KL, Prigozhin DM, Beatty PR, Harris E (2006). Murine model for dengue virus-induced lethal disease with increased vascular permeability. J Virol.

[248] Stein DA, Huang CY, Silengo S, Amantana A, Crumley S, Blouch RE (2008). Treatment of AG129 mice with antisense morpholino oligomers increases survival time following challenge with dengue 2 virus. J Antimicrob Chemother.

[249] Kyle JL, Balsitis SJ, Zhang L, Beatty PR, Harris E (2008). Antibodies play a greater role than immune cells in heterologous protection against secondary dengue virus infection in a mouse model. Virology.

[250] Prestwood TR, Prigozhin DM, Sharar KL, Zellweger RM, Shresta S (2008). A mouse-passaged dengue virus strain with reduced affinity for heparan sulfate causes severe disease in mice by establishing increased systemic viral loads. J Virol.

